# Development of a telomere vector‐based approach to overcome limitations caused by lethal phenotypes in the study of essential genes in *Magnaporthe oryzae*

**DOI:** 10.1111/mpp.13460

**Published:** 2024-05-02

**Authors:** Louisa Wirtz, Florencia Casanova, Ulrich Schaffrath, Alex Wegner

**Affiliations:** ^1^ Department of Molecular Plant Physiology RWTH Aachen University Aachen Germany

**Keywords:** CRISPR/Cas9, gene deletion, *Magnaporthe oryzae*, mutational analyses, reverse genetics, rice blast

## Abstract

Reverse genetic approaches are common tools in genomics for elucidating gene functions, involving techniques such as gene deletion followed by screening for aberrant phenotypes. If the generation of gene deletion mutants fails, the question arises whether the failure stems from technical issues or because the gene of interest (GOI) is essential, meaning that the deletion causes lethality. In this report, we introduce a novel method for assessing gene essentiality using the phytopathogenic ascomycete *Magnaporthe oryzae*. The method is based on the observation that telomere vectors are lost in transformants during cultivation without selection pressure. We tested the hypothesis that essential genes can be identified in deletion mutants co‐transformed with a telomere vector. The *M. oryzae* gene *MoPKC*, described in literature as essential, was chosen as GOI. Using CRISPR/Cas9 technology transformants with deleted GOI were generated and backed up by a telomere vector carrying a copy of the GOI and conferring fenhexamid resistance. Transformants in which the GOI deletion in the genome was not successful lost the telomere vector on media without fenhexamid. In contrast, transformants with confirmed GOI deletion retained the telomere vector even in absence of fenhexamid selection. In the latter case, the maintenance of the telomere indicates that the GOI is essential for the surveillance of the fungi, as it would have been lost otherwise. The method presented here allows to test for essentiality of genes when no mutants can be obtained from gene deletion approaches, thereby expanding the toolbox for studying gene function in ascomycetes.

## Graphical abstract

By combining telomere vectors with the expression cassette for a gene of interest (GOI) and CRISPR/Cas9‐mediated gene deletion, the essentiality of the GOI can be tested by cultivation on non‐selective medium.

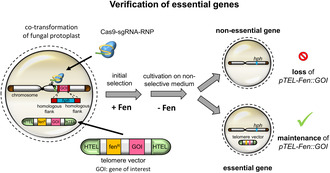

## 1 INTRODUCTION

Phytopathogenic fungi pose a threat to global food and raw material production, due to their potential to cause substantial economic losses. Despite progress in breeding for high‐yield crop varieties and the use of fungicides, plant pathogens continue to threaten crops because they evolve resistances to certain fungicide classes (Fischer & Edmeades, 2010; McDonald & Stukenbrock, 2016; Oerke, 2006). A silver bullet could identify essential genes in pathogens for targeted development of novel plant protection agents that would prevent the development of possible fungicide resistances (Cole, 2002).

In fungi, approximately one‐fifth of the genome consists of essential genes (Decottignies et al., 2003; Winzeler et al., 1999) and their identification is described as the primary task in genome‐based validation of potential drug targets (Chalker & Lunsford, 2002; Decottignies et al., 2003). Molecular genetic methods to identify and prove the essentiality of genes may involve the generation of conditional lethal mutants, for example, by point mutations (Chalker & Lunsford, 2002; Firon et al., 2002). An alternative is the activation or repression of gene expression using regulatable promoters (Cairns et al., 2016; Dichtl et al., 2012; Missall & Lodge, 2005; O'Meara et al., 2015; Roemer et al., 2003), but such promoters are potentially leaky and their applicability is restricted to certain organisms (Care et al., 1999). In *Aspergillus fumigatus*, insertional mutagenesis in combination with parasexual genetics has been used for the identification of essential genes (Firon et al., 2002, 2003; Ichinomiya et al., 2007). As this approach relies on a parasexual lifestyle, it is not universally applicable to other fungi. Other methods include the inhibition of protein activity, which is an elegant method but can only be applied to enzymes whose substrates are known and for which inactive analogues are available (Dang et al., 2011; Penn et al., 2015).

**FIGURE 1:**
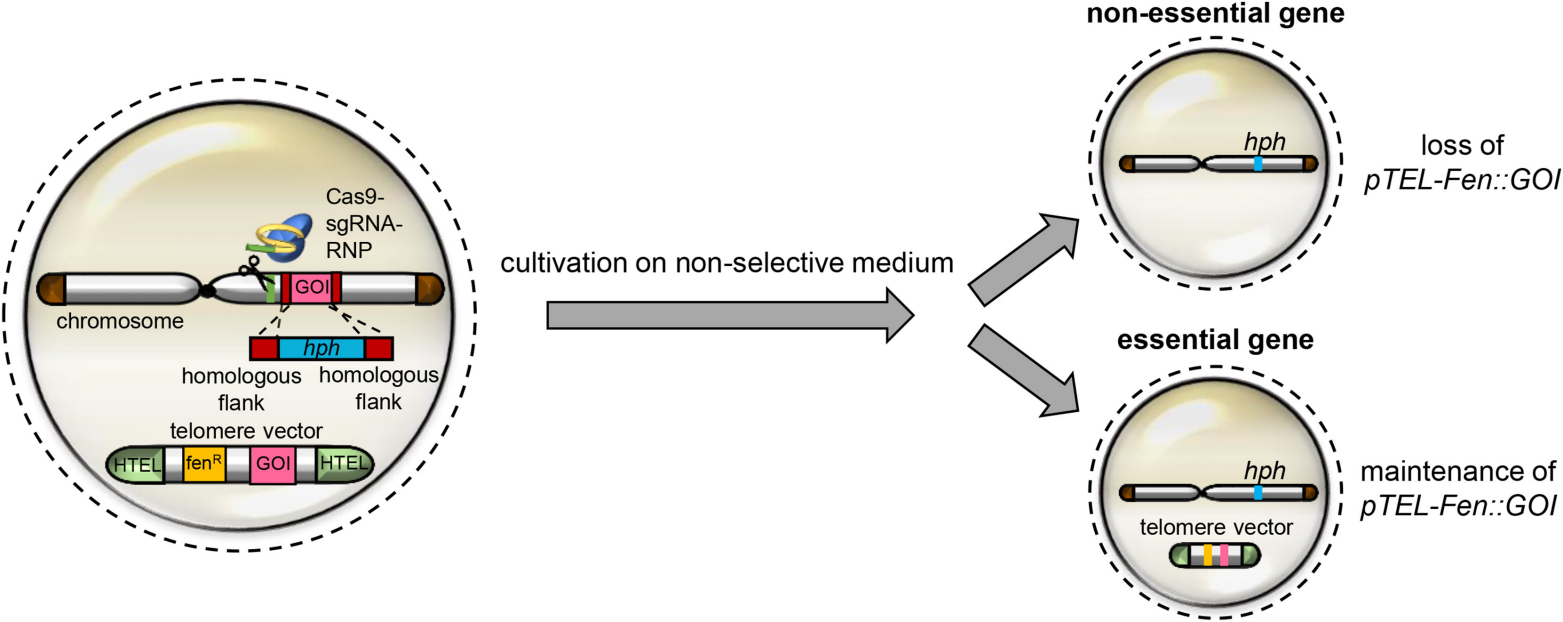
Schematic presentation of the strategy for identification of essential genes. Protoplasts are co‐transformed with a CRISPR/Cas9‐sgRNA‐ribonucleoprotein (RNP)‐complex, a gene‐deletion construct (Hyg^R^‐RT) and the telomere vector *pTEL‐Fen::GOI*, encoding the gene of interest (GOI) under the control of a constitutive promoter. The RNP and Hyg^R^‐RT are used to perform targeted deletion of the GOI in the genome. A telomere vector encoding the same GOI and a fenhexamid‐resistant cassette is additionally co‐transformed. The telomere vector is only maintained in transformants during selection with fenhexamid and gets lost without selection pressure. However, if the GOI, provided on the telomere vector and deleted in the genome, is essential for the viability of the fungus, the telomere vector is stable in transformants even on medium without fenhexamid. Thereafter, testing of transformants for fenhexamid resistance will only be successful if the GOI is an essential gene and thus the telomere vector is maintained.

All of the above‐mentioned methods have certain limitations and do not provide a versatile tool for testing gene essentiality in most ascomycetes. We therefore aimed to develop a fast and efficient method using the model organism for plant‐pathogenic fungi *Magnaporthe oryzae*. The method is based on the co‐transformation of *M. oryzae* protoplasts using a CRISPR/Cas9‐sgRNA‐ribonucleoprotein (RNP)‐complex together with a DNA deletion construct for targeted gene‐replacement of the genomic version of the GOI and a transiently stable telomere vector containing a copy of the GOI. Telomere vectors contain telomeric sequences that prevent them from integrating into the host genome at high rates and replicate autonomously as centromere‐free mini‐chromosomes (Barreau et al., 1998; Javerzat et al., 1993). Previous studies have shown that plasmids containing a pair of human telomeres can be efficiently used to transform filamentous fungi and that these vectors are only stable in host cells under selection conditions (Barreau et al., 1998; Leisen et al., 2020; Perrot et al., 1987). However, the stability varies between different fungi and the use of telomere vectors prevents genomic integration, ranging from 85% in *Botrytis cinerea* to 100% in *Podospora anserina* (Barreau et al., 1998; Leisen et al., 2020). By combining the strengths of CRISPR/Cas9 technology with telomere vectors, marker‐free genome editing can be achieved, as it has been reported for the fungi *B. cinerea* and *M. oryzae* (Leisen et al., 2020). While telomere vectors are lost at high percentages under nonselective growth conditions, we speculated that such a vector would be maintained if it encodes an expression cassette for an essential gene that is simultaneously deleted during the transformation process in the genome (Figure 1). To test this hypothesis, the *M. oryzae* gene *MoPKC* was chosen; this encodes a protein kinase C, an enzyme belonging to the serine/threonine kinase family, that is found in all eukaryotes (Penn et al., 2015). The *Mo*Pkc protein is involved in signalling related to growth, development and cell death and has been reported as a gene essential for the viability of *M. oryzae* (Penn et al., 2015). Results presented here indicate that the telomere vector carrying the *MoPKC* gene did not get lost, even under nonselective conditions, in mutants in which the same gene was simultaneously deleted on the chromosome. Our findings were further exemplified by using the *MoALB1* gene, whose deletion results in a whitish phenotype and serves as an easily traceable marker for assessing the loss or maintenance of telomere vectors.

## 2 RESULTS

The approach towards establishing a novel tool for the study of essential genes in ascomycetes encompassed two consecutive steps: the first to demonstrate that telomere vectors get lost during cultivation of transformants without selection pressure over a considerable time period, and the second to show that such a vector is maintained when it encodes an essential gene that has simultaneously been deleted in the genome of the target organism. As a case study, we chose *M. oryzae*, a well‐established model organism for plant‐pathogenic fungi, and selected two functionally characterized genes. The loss of one of these genes could be easily traced back due to a colour‐changed phenotype (*MoALB1*) and the second encodes a protein kinase C (*Mo*Pkc) known to be essential for viability of the fungus.

### 2.1 Telomere vectors get lost without selection pressure

**FIGURE 2:**
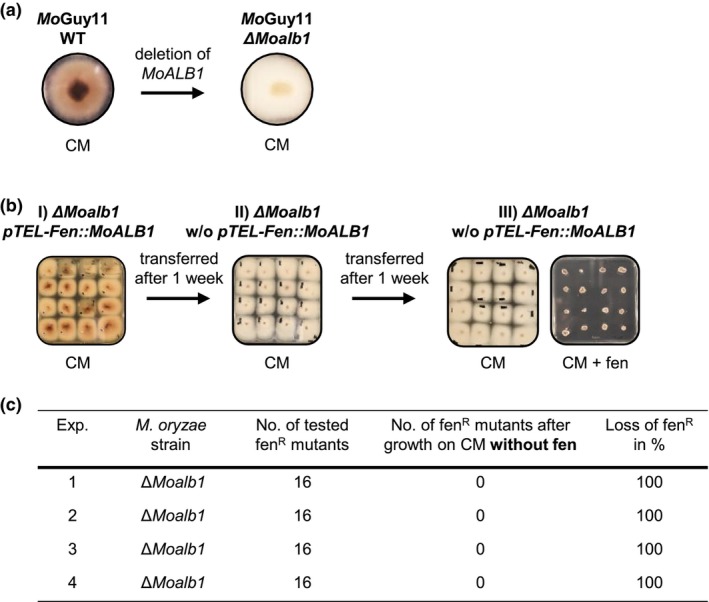
Loss of *pTEL‐Fen::MoALB1* in the absence of selection pressure. (a) While mycelium of *Magnaporthe oryzae* isolate Guy11 is brownish, the deletion of *MoALB1* results in a whitish phenotype. (b) Protoplasts of *Moalb1* mutant were transformed with the telomere vector *pTEL‐Fen::MoALB1*. After initial selection with fenhexamid (fen), Δ*Moalb1::pTEL‐Fen::MoALB1* transformants were cultivated for 2 weeks on nonselective complete medium (CM). After 1 week (colony diameter 2 cm), mycelium from the border of a colony was transferred to new CM plate. While the mycelium of the transformants appeared brown immediately after selection (I), it became white without selection pressure (II), indicating the loss of the telomere vector *pTEL‐Fen::MoALB1*. After cultivation without selection pressure for 2 weeks, transformants were transferred to CM or CM + fen (III). Tested transformants grew with whitish mycelium on CM and no growth occurred on fenhexamid‐containing medium, both confirming the loss of the telomere vector *pTEL‐Fen::MoALB1*. (c) In four independent experiments, 16 fen^R^ transformants were analysed with regard to fenhexamid sensitivity or resistance after cultivation on nonselective medium for 2 weeks. In each experiment, all of the 16 transformants lost fenhexamid resistance, that is, the telomere vector *pTEL‐Fen::MoALB1*.

Mycelium of wild‐type *M. oryzae* isolate Guy11 appears brownish when grown on complete medium (CM). Deletion of the gene *MoALB1* (MGG_07219), which encodes a polyketide synthase that is involved in melanin biosynthesis, results in a whitish appearance of the mycelium (Foster et al., 2018) (Figure 2a). We transformed protoplasts of a whitish mutant (*∆Moalb1*) with the telomere vector *pTEL‐Fen::MoALB1*, which carries the wild‐type *MoALB1* gene under the control of the constitutive *Prbp27* promoter and a gene conferring resistance to fenhexamid (fen) (Wegner et al., 2022). After initial selection with fen, mutants were transferred to nonselective CM. The brownish colour of the mycelium of these mutants indicated the complementation of ∆*Moalb1* with the wild‐type gene provided on the telomere vector. After 1 week of cultivation on CM without fen, the mutant colonies had a brownish centre and whitish hyphae at the outer border, corresponding to the oldest and youngest parts of a growing colony, respectively (Figure 2b‐I). We concluded that the telomere vector, with the wild‐type *MoALB1* gene, had got lost in the youngest parts of the colonies during cultivation for 1 week. To test this hypothesis, we transferred white mycelium from the border of 16 colonies to CM without fen and cultivated it for another week. The entire mycelium of all 16 colonies had a whitish colour (Figure 2b‐II), confirming that the telomere vector with the wild‐type *MoALB1* gene was lost. To further corroborate this conclusion, colonies from the latter plate were transferred to either CM or CM + fen and cultivated for another week (Figure 2b‐III). While colonies on CM plates grew with a whitish mycelium, no growth was observed on CM + fen. This unequivocally showed that the telomere vector (*pTEL*‐*Fen::MoAlb1*), which also carried the gene conferring fen resistance, had been lost during cultivation on CM without fen. We performed four independent replicates of the above‐described transformation experiment and selected in each case 16 mutants with fen (Figure 2c). Cultivation of these 16 mutants on CM without fen resulted in the loss of fen resistance, at a rate of 100%. With these results the first requirement for the proposed assay, the loss of telomere vectors without selection pressure, was fulfilled.

### 2.2 Co‐transformation with *pTEL*‐*Fen::MoPKC* enables deletion of the essential gene *MoPKC*

Next, we selected *MoPKC*, a gene reported in the literature to be essential for growth of *M. oryzae* (Penn et al., 2015), as candidate for the proof‐of‐concept experiments. In this assay, we aimed to delete the genomic copy of *MoPKC* and concurrently provide the sequence of the wild‐type gene, in an analogous approach as described above for *MoALB1*, on a telomere vector (*pTEL*‐*Fen::MoPKC*). The targeted deletion was achieved by inducing a DNA double‐strand break (DSB) upstream of the gene *MoPKC* using a CRISPR/Cas9‐RNP as described in Leisen et al. (2020). By targeting this region upstream of the gene, the Cas9‐endonuclease was kept from cleaving the *MoPKC* gene copy provided on the telomere vector. For transformation of *M. oryzae* protoplasts, the Cas9^4×SV40^ was mixed with the sgRNA and a template containing the hygromycin (hyg) resistance‐encoding gene *hph* for replacement of the genomic version of *MoPKC*.

**FIGURE 3:**
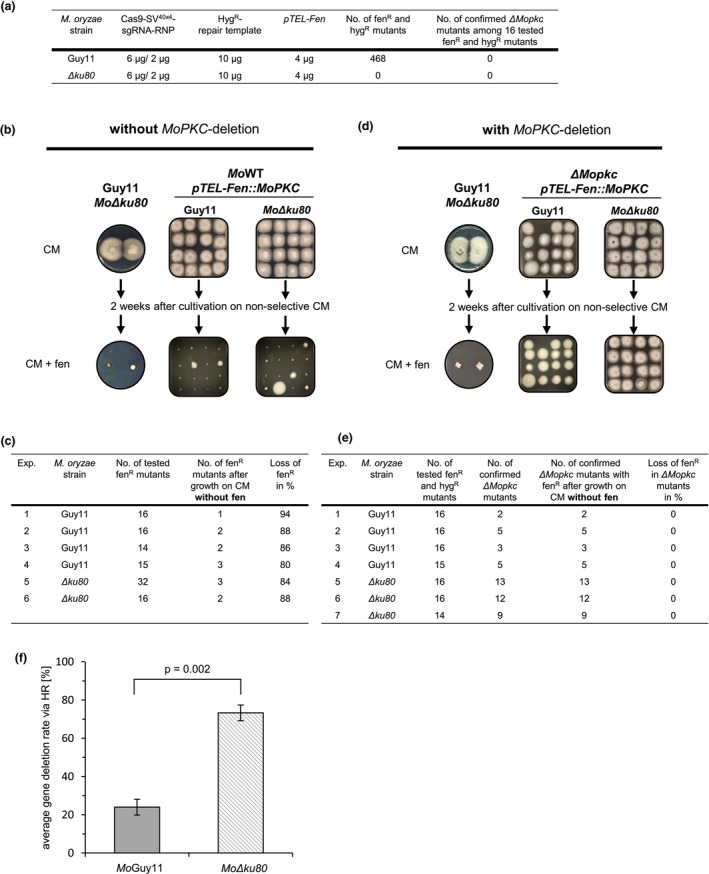
Co‐transformation of *pTEL‐Fen::MoPKC* enables deletion of the essential gene *MoPKC* in the genome. (a) Guy11 and *Mo*Δ*ku80* protoplasts were co‐transformed with a Cas9‐sgRNA‐RNP targeting the native locus of *MoPKC*, a Hyg^R^‐RT and the telomere vector *pTEL‐Fen*. When using *Magnaporthe oryzae* Guy11, 468 transformants were generated, but for none of them deletion of *MoPKC* was confirmed. When using the nonhomologous end‐joining‐deficient mutant *Mo*Δ*ku80*, no fenhexamid‐resistant (fen^R^) transformants were generated. (b) Transfer of the wild‐type strain Guy11 and the mutant *Mo*Δ*ku80* from complete medium (CM) to CM + fen indicated that they are not resistant to this fungicide. Protoplasts of *M. oryzae* Guy11 and *Mo*Δ*ku80* were transformed with the telomere vector *pTEL‐Fen::MoPKC* but without deletion of *MoPKC* on the genome. Sixteen fen^R^ Guy11 or *Mo*Δ*ku80* transformants were initially transferred to CM + fen, followed by cultivation on CM without fen for 2 weeks and then transferred again to CM + fen. (c) Across four independent experiments, 14–16 fen^R^ Guy11 transformants were tested for fen‐sensitivity/resistance after cultivation on CM without fen for 2 weeks. For *Mo*Δ*ku80*, 16–32 fen^R^ transformants were analysed in two independent experiments. On average, 87% of the tested Guy11 transformants and 86% of the *Mo*Δ*ku80* transformants lost fenhexamid resistance, along with the telomere vector *pTEL‐Fen::MoPKC*, after cultivation on CM without fen. (d) Protoplasts of *M. oryzae* Guy11 and *Mo*Δ*ku80* were co‐transformed with the Cas9‐sgRNA‐RNP, the Hyg^R^‐repair template and the telomere vector *pTEL‐Fen::MoPKC*. After initial selection with fen and hygromycin (hyg), 14–16 fen^R^ + hyg^R^ Guy11 and *Mo*Δ*ku80* transformants were transferred to CM + fen, and subsequently cultivated on CM without fen for 2 weeks and then retransferred to CM + fen. All confirmed Δ*Mopkc* transformants were able to grow on the selective medium. (e) Fifteen or 16 fen^R^ + hyg^R^ Guy11 transformants were tested in quadruplicate for deletion of the genomic version of the *MoPKC* gene and fen resistance after 2 weeks of cultivation on CM without fen. In mutants in which PCR analysis confirmed deletion of *MoPKC* by homologous recombination, no loss of fen resistance had occurred, meaning that the telomere vector *pTEL‐Fen::MoPKC* was still present. In three independent experiments, 14–16 fen^R^ + hyg^R^ transformants of *Mo*Δ*ku80* were analysed for *MoPKC* deletion and resistance to fen. Consistent with findings for Guy11, mutants with confirmed *MoPKC* deletion did maintain the telomere vector *pTEL‐Fen::MoPKC* and thus fen resistance. (f) Average deletion rates via homologous recombination in Guy11 and *Mo*Δ*ku80*. Values are calculated as ratio of transformants with confirmed *MoPKC* deletion relative to all tested fen^R^ + hyg^R^ transformants (Figure 4e). Bars display the average and standard deviation calculated from four and three independent experiments for Guy11 and *Mo*Δ*ku80*, respectively. The level of significance was determined using *t* test.

At first, we deleted *MoPKC* in the wild‐type Guy11 isolate and added a telomere vector with a gene cassette providing fen resistance but without the *MoPKC* gene (*pTEL‐Fen*). Co‐selection on hyg and fen‐containing medium resulted in a total of 468 mutants (Figure 3a). However, PCR analysis and sequencing of the *MoPKC* locus for selected mutants revealed for none of them homologous integration of the hygromycin‐resistance cassette at the *MoPKC* wild‐type locus. For improvement, we did the same transformation in the *Mo*Δ*ku80* genotype, a mutant known to be impaired in nonhomologous end‐joining (NHEJ) (Villalba et al., 2008). In this case, no fen‐resistant transformants were obtained when using the above‐mentioned telomere vector without the *MoPKC* gene (*pTEL‐Fen*) (Figure 3a). This led us to speculate that in the case of the *Mo*Δ*ku80* genotype, deletion of the genomic version of the *MoPKC* gene has occurred, that is, homologous integration of the hygromycin‐resistance cassette was successful, and thus mutants died.

Because interpretations drawn from such indirect evidence are challenging to trace back, we next conducted an analogous experiment with a telomere vector carrying the *MoPKC* gene (*pTEL‐Fen::MoPKC*). Firstly, we verified that neither Guy11 nor *Mo*Δ*ku80* were able to grow on CM + fen, which was in accordance with Wegner et al. (2022) (Figure 3b). Next both genotypes, Guy11 and *Mo*Δ*ku80*, were transformed with *pTEL‐Fen::MoPKC* but in the absence of CRISPR/Cas9, meaning that the *MoPKC* gene will not be deleted in the genome (Figure 3b). Mutants that had taken up the telomere vector were initially selected on CM + fen, transferred to CM without fen and further cultivated for 2 weeks. Thereafter, mutants were rescreened on CM + fen (Figure 3b). Almost all mutants did not survive fen selection, indicating the loss of the telomere vector during cultivation without selection pressure. This loss occurred with a frequency of 80%–94% or 84%–88% in the Guy11 and *Mo*Δ*ku80* genotypes, respectively (Figure 3c). For the few mutants that survived the final selection, it must be concluded that the fen‐resistance cassette had integrated into the genome. Next, both *M. oryzae* genotypes were co‐transformed with *pTEL‐Fen::MoPKC*, the Cas9‐sgRNA‐RNP and a gene deletion construct for the genomic version of *MoPKC*. Again, an initial selection of transformants was done on fen‐containing medium and selected mutants were tested by sequencing of respective PCR products for deletion of *MoPKC* in the genome (Figure 3d). In four independent biological replicates for Guy11, the gene deletion rate varied from 2/16 to 5/15 and for the *Mo*Δ*ku80* genotype from 13/16 to 9/14. This highlighted the significantly better gene deletion rate for the *M. oryzae* genotype *Mo*Δ*ku80*, with disabled NHEJ, compared to Guy11 at 73% and 24%, respectively (Figure 3f). The mutants with confirmed deletion of *MoPKC* in the genome were cultivated further for 2 weeks on CM without fen and thereafter rescreened on CM + fen (Figure 3d). None of these mutants lost the ability to grow on fen‐containing medium (Figure 3e), indicating the maintenance of the telomere vector even in absence of selection pressure. In other words, these results demonstrate that in mutants where the *MoPKC* gene has successfully been deleted in the genome, an otherwise instable telomere vector is maintained when it carries an expression cassette for the same gene.

## 3 DISCUSSION

Telomere vectors in combination with the CRISPR/Cas9 technology have already been reported as powerful tools to achieve marker‐free genome editing in *M. oryzae* (Leisen et al., 2020). Building on these results, a *pTEL‐Fen* and CRISPR/Cas9‐mediated strategy for verification of essential genes in ascomycetes was established in this study. The basis for this strategy is the observation that telomere vectors conferring fen resistance get lost after transformation under nonselective growth conditions, as shown for *M. oryzae ∆Moalb1* (Figure 2). This mutant was particularly useful because it has a whitish mycelium that could be complemented with a telomere vector containing the wild‐type *MoALB1* gene and thus mycelium colour changed to the wild‐type (brown) phenotype. The loss of the telomere vector was easily visible by monitoring the occurrence of colonies with whitish mycelium in the absence of fen selection. To demonstrate that such a vector becomes stable when it contains a functional copy of an essential gene, we performed a co‐transformation assay in which the deletion of GOI (*MoPKC*) on the chromosome and the substitution with a GOI copy on a telomere vector was achieved simultaneously.

The respective telomere vector (*pTEL‐Fen::MoPKC*) was lost in at least 86% of the selected transformants after 2 weeks of cultivation on nonselective medium when the gene was not simultaneously deleted in the genome (Figure 3). This underpins that fungal genes on telomere vectors do not integrate into the genome, or at least are prevented from doing so at high percentages, thus supporting similar findings reported by Perrot et al. (1987), Barreau et al. (1998) and Leisen et al. (2020). Co‐transformation of the *M. oryzae* isolate Guy11 and the *Mo∆ku80* mutant with *pTEL‐Fen::MoPKC*, the in vitro assembled CRISPR/Cas9‐sgRNA complex and the *MoPKC*‐deletion construct resulted in a high rate of transformants similar to that described in Leisen et al. (2020). PCR analysis of selected transformants revealed significantly higher rates of homologous integration, resulting in deletion of *MoPKC* on the chromosome, in the NHEJ‐deficient mutant *Mo∆ku80* (Figure 3a). These high editing rates were achieved with microflanks for homologous recombination, as described by Foster et al. (2018), which reconfirmed the improvement by avoiding cloning of 1000 bp long homologous flanks. Transformants with a deletion of *MoPKC* in the genome maintained the telomere vector *pTEL*‐*Fen::MoPKC*, even if cultivated on nonselective medium (CM without fen) for several weeks (Figure 3e). Conversely, when co‐transforming *M. oryzae* protoplasts with *pTEL‐Fen::MoPKC* but without *MoPKC*‐deletion construct, the telomere vector was not maintained but rather got lost after a few weeks (Figure 3b,c). However, due to the low rate of genome integration observed with telomere vectors, the approach should always include a concurrent PCR analysis to confirm the fate of the telomere vector. In accordance with the study of Penn et al. (2015), these results emphasize the essential role of *MoPKC* for the viability of *M. oryzae*. Furthermore, our strategy of using a *pTEL‐Fen* and CRISPR/Cas9‐mediated genome editing to verify essential genes in ascomycetes has thus been successfully established. It should be noted that the use of the constitutive promoter *Prbp27* might cause problems if the native gene expression pattern significantly differs from that of the constitutive promoter. In this case, the use of the native promoter sequence of the respective gene should be considered. Consequently, it would be necessary to target a sequence with the Cas‐9‐RNP in the sequence upstream of the promoter or downstream of the terminator region. However, when dealing with gene dosage effects, it should also be carefully considered that the copy number of the telomere vector was determined to be lower than the chromosomal copy number, at least in *B. cinerea* (Leisen et al., 2020), which could also have an impact on the gene dosage effect. Apart from *M. oryzae*, the telomeric nucleotide sequence (T_2_AG_2_)_n_ is conserved in many other filamentous fungi such as *Fusarium oxysporum*, *Podospora anserina*, *Cladosporium fulvum*, *Aspergillus nidulans* and *Neurospora crassa* (Bhattacharyya, 1997; Coleman et al., 1993; Farman & Leong, 1995; Javerzat et al., 1993; Powell & Kistler, 1990; Schechtman, 1990). In addition, high transformation frequencies and autonomous replication of linear DNA molecules with telomeric ends have already been achieved in *F. oxysporum*, *Histoplasma capsulatum*, *Nectria haematococca*, *Cryprococcus neoformans*, *P. anserina* and *B. cinerea* (Kistler & Benny, 1992; Powell & Kistler, 1990; Woods & Goldman, 1992). Based on these findings, the combination of efficient CRISPR/Cas9‐technology with non‐integrative vectors containing human telomeres (telomere vectors) represents a promising approach for verification of essential genes, not only in *M. oryzae*, but also in perspective, in filamentous fungi in general. This provides a strategy to determine whether the absence of mutants in a gene deletion approach is due to the essential role of the target gene or to a failure of gene replacement.

## 4 EXPERIMENTAL PROCEDURES

### 4.1 Fungal isolates of *M. oryzae*

*M. oryzae* Guy11 and the *Mo*Δ*ku80* mutant were provided by D. Tharreau from CIRAD (Montpellier, France) and were cultivated on complete medium (CM), supplemented with agar, at a temperature of 23°C in absence of light (Talbot et al., 1993).

### 4.2 Vector construction

The vector *pTEL*‐Fen‐MCS (File S1), which was used as starting vector in this study, was obtained by cloning the expression cassette of pTK144‐MCS (P*rbp27‐*MCS‐Stop‐mRFP‐T*tubB*) (pTK144 provided by Thomas Kroj, INRA, UMR BGPI, Montpellier, France) into the telomere vector *pTEL*‐*Fen* (provided by Matthias Hahn, TU, Kaiserslautern, Germany) using Gibson assembly (Gibson Assembly Master Mix; New England BioLabs). Therefore, we amplified the DNA sequence of the expression cassette from the plasmid pTK144‐MCS by PCR, using Phusion Hot Start High Fidelity DNA polymerase (Thermo Fisher Scientific) and the primer pair pTK144‐OE_for/rev (Table S1). Next, the PCR product of the overexpression construct was purified using the NucleoSpin gel and PCR clean‐up kit (Macherey‐Nagel). The vector was restricted with BamHI/KpnI (fast digest enzymes, Thermo Fisher Scientific) and then the overexpression cassette was inserted using Gibson assembly. Afterwards, the ligated plasmid (*pTEL*‐*Fen*‐MCS) was used for transformation of *Escherichia coli* DH5‐α (Thermo Fisher Scientific). The vector was purified from *E. coli* using the NucleoSpin Plasmid Kit (Macherey‐Nagel) and verified by sequencing (Microsynth SeqLab, Göttingen, Germany).

To generate the vectors *pTEL*‐*Fen::MoALB1* and *pTEL*‐*Fen::MoPKC* (File S1), the previously generated telomere vector *pTEL*‐*Fen*‐MCS was digested with BcuI and KpnI. The coding sequences of *MoPKC* (MGG_08689) and *MoALB1* (MGG_07219) were amplified by PCR from genomic DNA of *M. oryzae* wild‐type isolate Guy11 using Phusion Hot Start High Fidelity DNA polymerase and the primer pairs PKC_MCS‐for/rev and ALB_MCS‐for/rev, respectively. Primers were designed with respective overlapping sequence to *pTEL*‐*Fen‐*MCS (Table S1). The PCR products were purified using the NucleoSpin Gel and PCR clean‐up kits and cloned into the BcuI/KpnI‐restricted telomere vector *pTEL‐Fen‐*MCS by Gibson assembly. Through this step, the genes were set under the control of the constitutive expression promoter P*rbp27*. The ligated vector was transformed into *E. coli*. Vectors were purified from *E. coli* using the NucleoSpin plasmid kit and successful insertion of the targeted genes into *pTEL‐Fen* was confirmed by sequencing.

### 4.3 Generation of Δ*Moalb1* mutant and complementation with *pTEL*‐*Fen::MoALB1*

Mutants of *M. oryzae* wild‐type isolate Guy11 and *Mo*Δ*ku80* were generated by standard protoplast transformation as described in Leisen et al. (2020) and Wegner et al. (2022). To target *MoALB1* by CRISPR/Cas9‐RNP, a specific single‐guide RNA (sgRNA) was synthesized as described in Leisen et al. (2020) and Wegner et al. (2022). Oligonucleotides used for synthesis of sgRNA (gRNA_MoALB1/gRNA_konst.) are listed in Table S1. The Δ*Moalb1* mutant was generated by mixing 120 μL of the protoplast suspension of *M. oryzae* isolate Guy11 with 6 μg Cas9‐RNP targeting *MoALB1* and 6 μg repair template (RT) suspended in a total volume of 60 μL Tris‐CaCl_2_ buffer. The RT encodes the hygromycin‐resistance gene *hph* and 50 bp‐long flanks to promote integration into the targeted gene locus by homologous recombination. These flanks were designed to be sequence‐identical to the targeted integration locus in the genome of *M. oryzae* (Foster et al., 2018). Fusion of the homologous flanks to *hph* was achieved by PCR using the primer pair MH‐ALB1_F/MH_ALB1_R. After transformation, protoplasts were poured into CM agar supplemented with 1.2 M sucrose and after 24 h a second layer of top agar containing 500 mg/mL hygromycin was added. Transformants that had grown through the top agar and exhibited a whitish phenotype were verified by PCR analysis for the insertion of Hyg^R^‐RT into the *MoALB1* locus.

For complementation of Δ*Moalb1*, protoplasts of Δ*Moalb1* mutant were transformed with 4 μg of the telomere vector *pTEL‐Fen::MoALB1*. Transformants were selected with CM containing 30 mg/mL fen and transferred to CM without fen immediately after they had grown through the top agar.

### 4.4 Transformation of *M. oryzae* Guy11 and *MoΔku80* using *pTEL*‐*Fen::MoPKC* with and without *MoPKC* deletion

**FIGURE 4:**
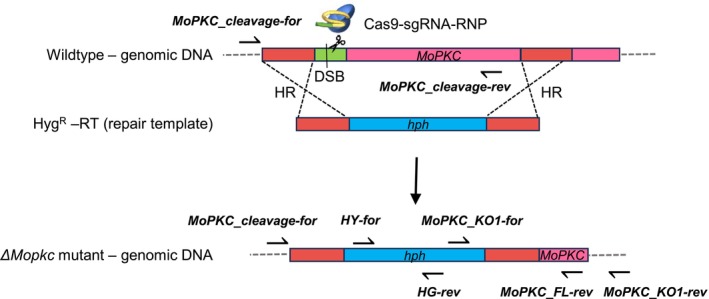
PCR‐based approach to confirm *MoPKC* gene deletion. Schematic setup of *MoPKC*‐gene‐deletion by using CRISPR/Cas9‐mediated genome editing and homologous recombination (HR) to replace *MoPKC* by the resistance marker gene *hph*. Because *MoPKC* deletion is lethal, *MoPKC* must be simultaneously provided on a telomere vector (not shown) to generate *Magnaporthe oryzae* transformants. A double‐strand break (DSB) was induced upstream of the *MoPKC* gene by the Cas9‐sgRNA‐ribonucleoprotein (RNP). By targeting the sequence upstream of *MoPKC*, the Cas9‐endonuclease is prevented from cutting the copy of *MoPKC* provided on the telomere vector. The DSB caused by the Cas9‐endonuclease facilitates the integration of the Hyg^R^‐repair template (RT) into the genome through HR, resulting in the deletion of *MoPKC*. A nucleotide sequence of 94 bp at the 3′‐end of the gene will be maintained. In order to verify HR between *MoPKC* and Hyg^R^‐RT, primer combinations MoPKC_cleavage‐for/rev, MoPKC_cleavage‐for/HG‐rev, HY‐for/MoPKC_FL‐rev and MoPKC_KO1‐for/rev were used for PCR analysis. Only if all four PCR products were amplified with calculated product sizes was the respective mutant considered as a successful gene deletion mutant.

Generation of *M. oryzae* Guy11 Δ*Mopkc::pTEL‐Fen::MoPKC* and *Mo*Δ*ku80/*Δ*Mopkc::pTEL‐Fen::MoPKC* mutants was achieved by co‐transformation of fungal protoplasts. Therefore, protoplasts of Guy11 and *Mo*Δ*ku80* were transformed with 6 μg of Cas9‐sgRNA‐RNP, 4 μg of the telomere vector *pTEL‐Fen::MoPKC* and 10 μg of the Hyg^R^‐RT. To prevent the Cas9‐sgRNA‐RNP from cutting the *MoPKC* gene provided on the telomere vector, the genomic region for the DSB was targeted upstream of *MoPKC* using a specific sgRNA. This region is not present on *pTEL‐Fen::MoPKC*. The sgRNA was synthesized using primers gRNA‐MoPKC/gRNA‐konst. as described in Leisen et al. (2020). Similar to the generation of the Δ*Moalb1* mutant, the hygromycin‐resistance gene *hph* was used as replacement for the *MoPKC* gene (Hyg^R^‐RT). Using primers MH‐PKC‐for/−rev, 50 bp‐long flanks sequence‐identical to the targeted gene locus were fused to *hph*. Transformants were selected with CM containing 30 mg/mL fen and 500 mg/mL hyg. Fenhexamid‐ and hygromycin‐resistant mutants were analysed by PCR for homologous recombination of the Hyg^R^‐RT into the *MoPKC* locus using primer pairs MoPKC_cleavage‐for/rev, MoPKC_cleavage‐for/HG‐rev, HY‐for/MoPKC_FL‐rev and MoPKC_KO1‐for/rev. Only those mutants, in which integration of the Hyg^R^‐RT via homologous recombination was confirmed, were further investigated (Figure 4).

### 4.5 Growth experiments on CM with and without fen

After initial selection, Δ*Moalb1::pTEL‐Fen::MoALB1* mutants were immediately transferred to CM without fen and cultivated 2 weeks with weekly transfer to new plates. Changes in phenotype (colour of mycelium) were documented photographically. After cultivation on nonselective CM without fen for 2 weeks, mutants were transferred to CM containing 30 mg/mL fen (CM + fen). Results were documented photographically after 7 days.

After mutants (Guy11 *pTEL‐Fen::MoPKC*, Guy11 Δ*Mopkc::pTEL‐Fen::MoPKC*, *Mo*Δ*ku80::pTEL‐Fen::MoPKC* and *Mo*Δ*ku80/*Δ*Mopkc::pTEL‐Fen::MoPKC*) had grown through the top agar with fen, they were further cultivated on CM containing 30 mg/mL fen for a few days and then transferred to CM without fen, to prevent integration of the telomere sequences into the genome. After 2 weeks of cultivation on nonselective CM, the mutants were transferred to CM containing 30 mg/mL fen. The results were documented photographically after 7 days.

## CONFLICT OF INTEREST STATEMENT

The authors declare that they have no competing interests.

## Supporting information


Data S1.

Table S1.

## Data Availability

The data that support the findings of this study are available from the corresponding author upon reasonable request.
