# Supplementary material for: Development of a telomere vector‐based approach to overcome limitations caused by lethal phenotypes in the study of essential genes in Magnaporthe oryzae
Source: Mol Plant Pathol. 2024 May 2;25(5):e13460. doi: 10.1111/mpp.13460 (PMC11064798; doi:10.1111/mpp.13460)
Supplement: Supplementary file 1 — Data S1. [file MPP-25-e13460-s002.docx]

**Supplementary file 1: Plasmid sequences**

1. ***pTEL-Fen-MCS***
2. ***pTEL-Fen::MoALB1***
3. ***pTEL-Fen::MoPKC***
4. ***pTEL-Fen-MCS***


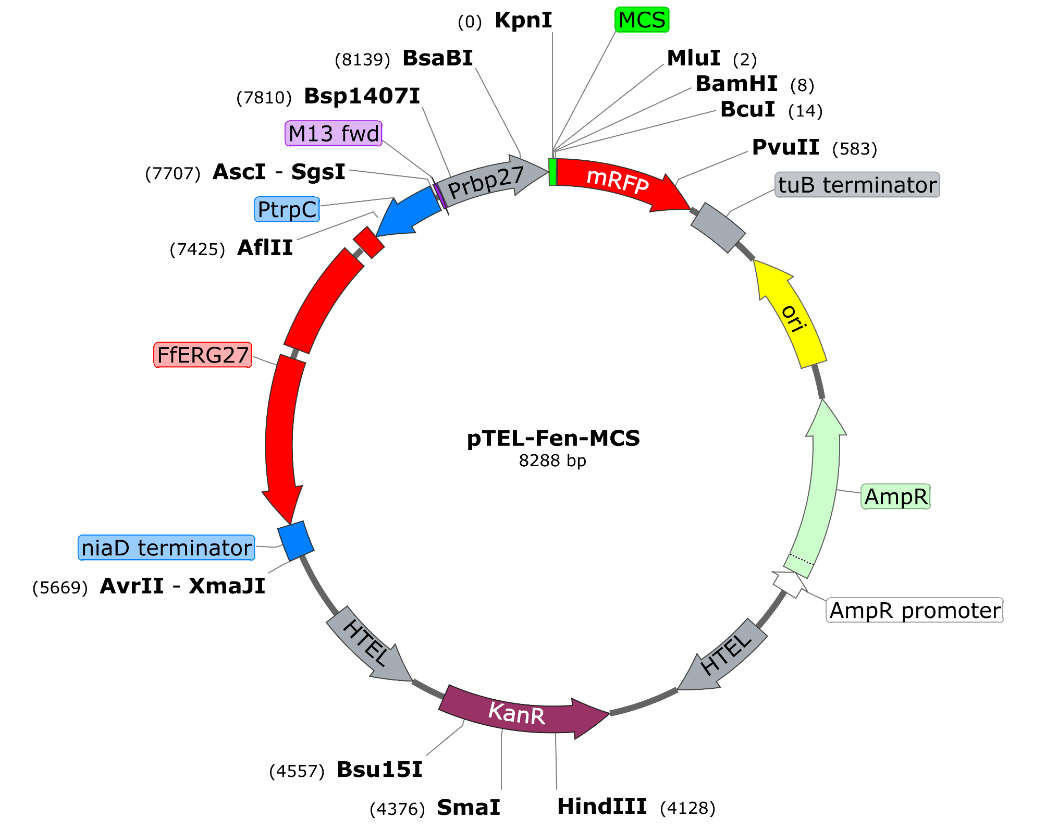


LOCUS pTELFen-MCS. 8288 bp DNA circular SYN 20-MAR-2024

DEFINITION synthetic circular DNA

ACCESSION .

VERSION .

KEYWORDS .

SOURCE synthetic DNA construct

ORGANISM synthetic DNA construct

REFERENCE 1 (bases 1 to 8288)

AUTHORS RiBa

TITLE -

JOURNAL -

COMMENT -

FEATURES Location/Qualifiers

source 1..8288

/mol_type="other DNA"

/organism="synthetic DNA construct"

misc_feature 23..697

/label=mRFP

misc_feature 740..973

/label=tuB terminator

rep_origin complement(1087..1675)

/direction=LEFT

/label=ori

/note="high-copy-number ColE1/pMB1/pBR322/pUC origin of

replication"

CDS complement(1846..2706)

/codon_start=1

/gene="bla"

/product="beta-lactamase"

/label=AmpR

/note="confers resistance to ampicillin, carbenicillin, and

related antibiotics"

/translation="MSIQHFRVALIPFFAAFCLPVFAHPETLVKVKDAEDQLGARVGYI

ELDLNSGKILESFRPEERFPMMSTFKVLLCGAVLSRVDAGQEQLGRRIHYSQNDLVEYS

PVTEKHLTDGMTVRELCSAAITMSDNTAANLLLTTIGGPKELTAFLHNMGDHVTRLDRW

EPELNEAIPNDERDTTMPAAMATTLRKLLTGELLTLASRQQLIDWMEADKVAGPLLRSA

LPAGWFIADKSGAGERGSRGIIAALGPDGKPSRIVVIYTTGSQATMDERNRQIAEIGAS

LIKHW"

promoter complement(2707..2811)

/gene="bla"

/label=AmpR promoter

misc_feature 3029..3521

/label=HTEL

CDS complement(3867..4682)

/label=KanR

misc_feature complement(4852..5342)

/label=HTEL

misc_feature 5672..5830

/label=niaD terminator

/note="3'"

CDS complement(join(5831..6643,6693..7220,7276..7362))

/codon_start=1

/label=FfERG27

/note="fferg27 (FFUJ_04356)"

/translation="MGSQLSPATAPWEGVSGQEQLFVLITGANSGIGLSIGERLIDEFL

ATRSLRSHLILIPTTRSKSKSLQTIQTLRGYANKAAQSSTALRSRAGSSYRWEDTIARI

HVLSLQLDLCDLRGVYSFANALLRGPVSNPEGLQGEYLRNVRIPRLDTVVFNAAYGGWS

GVNYPKAVWTILTQGLVQSVTWPNFKMALPTALLNEKRNYNYPKEPLLGEVFTACVFGH

YILAHELLPLLSRRSETETPGRLVWSSSLEAVDSVLDMSDFQCFNGKGPYESAKRVTDI

LSLTATLPAAMPSSSRFFTPDDPSEARDKPIRPRMYLTHPGIVASTLFPVPWFLMWAYE

LALLISRWIGSPWHNTDSYTGAKSPVWIALQEQSALDELGAERIKWGSSSNRHMQVEVK

KTEVEGWGWEGKVEDAAALEADTAVGVFRKTIGRKRGAKDVTKEDIVRFEELGAECWER

MENMRHEWETILRVKKA"

misc_feature complement(7363..7706)

/label=PtrpC

/note="PtrpC"

misc_feature 7743..8270

/label=Prbp27

misc_feature join(8272..8288,1..18)

/label=MCS

ORIGIN

1 cacgcgtgga tccactagtg ctgcctcctc cgaggacgtc atcaaggagt tcatgcgctt

61 caaggtgcgc atggagggct ccgtgaacgg ccacgagttc gagatcgagg gcgagggcga

121 gggccgcccc tacgagggca cccagaccgc caagctgaag gtgaccaagg gcggccccct

181 gcccttcgcc tgggacatcc tgtcccctca gttccagtac ggctccaagg cctacgtgaa

241 gcaccccgcc gacatccccg actacttgaa gctgtccttc cccgagggct tcaagtggga

301 gcgcgtgatg aacttcgagg acggcggcgt ggtgaccgtg acccaggact cctccctgca

361 ggacggcgag ttcatctaca aggtgaagct gcgcggcacc aacttcccct ccgacggccc

421 cgtaatgcag aagaagacaa tgggctggga ggcctccacc gagcggatgt accccgagga

481 cggcgccctg aagggcgaga tcaagatgag gctgaagctg aaggacggcg gccactacga

541 cgccgaggtc aagaccacct acatggccaa gaagcccgtg cagctgcccg gcgcctacaa

601 gaccgacatc aagctggaca tcacctccca caacgaggac tacaccatcg tggaacagta

661 cgagcgcgcc gagggccgcc acagtactgg agcatgaatc attccactca acattcaggc

721 tcctctgcgc acgtaaagtg ccaaaggcaa taccctgctc ggtggaatgc cgccgggctt

781 gtcgatttta cgcacatatg cgcattcttg acttgaagcg gaggagttct tcgttgcggg

841 ttacagtgtt ttaataaaag aatggtcaaa tcaaactgct agatatacct gtcagacact

901 ctagttgttg acccctatac tcttaataca tcagacagta catgcatgtt gcatgatgat

961 gatatgtctg tttagattcc agtgtctact gctggcctag tttctcggta ctatgcatac

1021 tacatgcatg tgagcaaaag gccagcaaaa ggccaggaac cgtaaaaagg ccgcgttgct

1081 ggcgtttttc cataggctcc gcccccctga cgagcatcac aaaaatcgac gctcaagtca

1141 gaggtggcga aacccgacag gactataaag ataccaggcg tttccccctg gaagctccct

1201 cgtgcgctct cctgttccga ccctgccgct taccggatac ctgtccgcct ttctcccttc

1261 gggaagcgtg gcgctttctc atagctcacg ctgtaggtat ctcagttcgg tgtaggtcgt

1321 tcgctccaag ctgggctgtg tgcacgaacc ccccgttcag cccgaccgct gcgccttatc

1381 cggtaactat cgtcttgagt ccaacccggt aagacacgac ttatcgccac tggcagcagc

1441 cactggtaac aggattagca gagcgaggta tgtaggcggt gctacagagt tcttgaagtg

1501 gtggcctaac tacggctaca ctagaaggac agtatttggt atctgcgctc tgctgaagcc

1561 agttaccttc ggaaaaagag ttggtagctc ttgatccggc aaacaaacca ccgctggtag

1621 cggtggtttt tttgtttgca agcagcagat tacgcgcaga aaaaaaggat ctcaagaaga

1681 tcctttgatc ttttctacgg ggtctgacgc tcagtggaac gaaaactcac gttaagggat

1741 tttggtcatg agattatcaa aaaggatctt cacctagatc cttttaaatt aaaaatgaag

1801 ttttaaatca atctaaagta tatatgagta aacttggtct gacagttacc aatgcttaat

1861 cagtgaggca cctatctcag cgatctgtct atttcgttca tccatagttg cctgactccc

1921 cgtcgtgtag ataactacga tacgggaggg cttaccatct ggccccagtg ctgcaatgat

1981 accgcgagac ccacgctcac cggctccaga tttatcagca ataaaccagc cagccggaag

2041 ggccgagcgc agaagtggtc ctgcaacttt atccgcctcc atccagtcta ttaattgttg

2101 ccgggaagct agagtaagta gttcgccagt taatagtttg cgcaacgttg ttgccattgc

2161 tgcaggcatc gtggtgtcac gctcgtcgtt tggtatggct tcattcagct ccggttccca

2221 acgatcaagg cgagttacat gatcccccat gttgtgcaaa aaagcggtta gctccttcgg

2281 tcctccgatc gttgtcagaa gtaagttggc cgcagtgtta tcactcatgg ttatggcagc

2341 actgcataat tctcttactg tcatgccatc cgtaagatgc ttttctgtga ctggtgagta

2401 ctcaaccaag tcattctgag aatagtgtat gcggcgaccg agttgctctt gcccggcgtc

2461 aacacgggat aataccgcgc cacatagcag aactttaaaa gtgctcatca ttggaaaacg

2521 ttcttcgggg cgaaaactct caaggatctt accgctgttg agatccagtt cgatgtaacc

2581 cactcgtgca cccaactgat cttcagcatc ttttactttc accagcgttt ctgggtgagc

2641 aaaaacagga aggcaaaatg ccgcaaaaaa gggaataagg gcgacacgga aatgttgaat

2701 actcatactc ttcctttttc aatattattg aagcatttat cagggttatt gtctcatgag

2761 cggatacata tttgaatgta tttagaaaaa taaacaaata ggggttccgc gcacatttcc

2821 ccgaaaagtg ccacctgacg tctaagaaac cattattatc atgacattaa cctataaaaa

2881 taggcgtatc acgaggccct ttcgtcttca agaattcgcg gccccgcatg ggcccagatc

2941 taggcctgca ggatgctagc ttcagacgtg tctagggata acagggtaat tcgaaccccc

3001 gcgccgcctt tgcgagggtg gagttgcctt agggttaggg ttagggttag ggttagggtt

3061 agggttaggg ttagggttag ggttagggtt agggtttagg gttagggtta gggttagggt

3121 tagggttagg gttagggtca gggtcagggg tagggtcagg ggtagggtca ggggtagggg

3181 taggggtagg gtcagggtta gggttagggt tagggttagg gttagggtta gggttagggt

3241 cagggttagg gttagggtta gggaggggta ggggtagggg tagggttagg gttagggtta

3301 gggttagggt tagggttagg gttagggtca gggtcagggt caggggtagg ggtaggggta

3361 ggggtagggg tagggttagg ggtagggtta ggggtagggg taggggtagg gttagggtta

3421 gggtttaggg ttagggttag ggttagggtt agggttaggg gttagggtta gggttagggt

3481 tagaaggtta gggttagggt tagggttaag ggttaagggt taggtgtggg gtgtggcgaa

3541 ttcctcgacc tgcaagcggc cgcttgcagg gggggggggg cgctgaggtc tgcctcgtga

3601 agaaggtgtt gctgactcat accaggcctg aatcgcccca tcatccagcc agaaagtgag

3661 ggagccacgg ttgatgagag ctttgttgta ggtggaccag ttggtgattt tgaacttttg

3721 ctttgccacg gaacggtctg cgttgtcggg aagatgcgtg atctgatcct tcaactcagc

3781 aaaagttcga tttattcaac aaagccgccg tcccgtcaag tcagcgtaat gctctgccag

3841 tgttacaacc aattaaccaa ttctgattag aaaaactcat cgagcatcaa atgaaactgc

3901 aatttattca tatcaggatt atcaatacca tatttttgaa aaagccgttt ctgtaatgaa

3961 ggagaaaact caccgaggca gttccatagg atggcaagat cctggtatcg gtctgcgatt

4021 ccgactcgtc caacatcaat acaacctatt aatttcccct cgtcaaaaat aaggttatca

4081 agtgagaaat caccatgagt gacgactgaa tccggtgaga atggcaaaag cttatgcatt

4141 tctttccaga cttgttcaac aggccagcca ttacgctcgt catcaaaatc actcgcatca

4201 accaaaccgt tattcattcg tgattgcgcc tgagcgagac gaaatacgcg atcgctgtta

4261 aaaggacaat tacaaacagg aatcgaatgc aaccggcgca ggaacactgc cagcgcatca

4321 acaatatttt cacctgaatc aggatattct tctaatacct ggaatgctgt tttcccgggg

4381 atcgcagtgg tgagtaacca tgcatcatca ggagtacgga taaaatgctt gatggtcgga

4441 agaggcataa attccgtcag ccagtttagt ctgaccatct catctgtaac atcattggca

4501 acgctacctt tgccatgttt cagaaacaac tctggcgcat cgggcttccc atacaatcga

4561 tagattgtcg cacctgattg cccgacatta tcgcgagccc atttataccc atataaatca

4621 gcatccatgt tggaatttaa tcgcggcctc gagcaagacg tttcccgttg aatatggctc

4681 ataacacccc ttgtattact gtttatgtaa gcagacagtt ttattgttca tgatgatata

4741 tttttatctt gtgcaatgta acatcagaga ttttgagaca caacgtggct ttcccccccc

4801 cccctgcaag cggccgcttg caggtcgagg aattcgccac accccacacc taacccttaa

4861 cccttaaccc taaccctaac cctaacccta accctaaccc taaccctaac ccctaaccct

4921 aaccctaacc ctaaccctaa ccctaaaccc taaccctaac cctaccccta cccctacccc

4981 taaccctacc cctaacccta cccctacccc tacccctacc cctacccctg accctgaccc

5041 tgaccctaac cctaacccta accctaaccc taaccctaac cctaacccta cccctacccc

5101 tacccctccc taaccctaac cctaaccctg accctaaccc taaccctaac cctaacccta

5161 accctaaccc taaccctgac cctaccccta cccctacccc tgaccctacc cctgacccta

5221 cccctgaccc tgaccctaac cctaacccta accctaaccc taaccctaac cctaaaccct

5281 aaccctaacc ctaaccctaa ccctaaccct aaccctaacc ctaaccctaa ccctaaccct

5341 aaggcaactc caccctcgca aaggcggcgc gggggttcga attaccctgt tatccctaga

5401 tacgtctgct ttttgttgac ttccattgtt cattccacgg acaaaaacag agaaaggaaa

5461 cgacagaggc caaaaagctc gctttcagca cctgtcgttt cctttctttt cagagggtat

5521 tttaaataaa aacattaagt tatgacgaag aagaacggaa acgccttaaa ccggaaaatt

5581 ttcataaata gcgaaaaccc gcgaggtcgc cgccccgtaa caaggcggat cgccggaaag

5641 gacccgcaaa tgataataat tatcaattcc taggctcact gatacatctg gcacctactt

5701 tataataata ctttattgaa atactttata gatactatat tcaaacatcc tctctcccat

5761 tattttagac gcagttcaat ggcaaaaact acaaccatat ctaaaccacc tctcagttac

5821 ttaaaacctc ctatgccttt ttaaccctaa gaatggtttc ccactcgtgt cgcatgttct

5881 ccatcctttc ccagcattcg gcgcccagtt cctcgaatct cacgatatct tccttggtca

5941 cgtctttagc gcctctcttt cgcccgatgg tcttcctgaa aacaccaaca gcggtgtccg

6001 cttcaagcgc cgccgcatcc tcgactttgc cctcccaacc ccagccttca acttctgtct

6061 tcttaacttc gacctgcatg tgacggttcg agctgctgcc ccacttgatg cgctctgcac

6121 ctagttcatc aagcgcagat tgctcttgga gcgcaatcca aacaggggat ttggcaccgg

6181 tatagctgtc tgtgttgtgc cagggcgaac caatccagcg actgatcaga agagcaagtt

6241 catatgccca catcagaaac cagggaactg ggaatagggt actggcaaca atacccggat

6301 gggtgagata catgcgcggt cgaatgggtt tatcgcgggc ctcgctgggg tcgtcggggg

6361 tgaagaagcg gctggaagat ggcatggctg cggggagagt tgcagtgagg gagagaatgt

6421 cggtgactcg tttagcggat tcgtacgggc ctttaccatt gaagcactgg aagtcggaca

6481 tatcaagaac actgtcgacg gcctcaagac tgctggacca gacaagacgg ccgggtgttt

6541 cggtttcgga tcgccgacta agtagtggaa gaagttcatg ggccaatata taatgaccaa

6601 aaacacaggc tgtaaacacc tcgccgagta aaggttcctt gggctgcatt cagtcaatgg

6661 gtgaaatcat tgagttactg cgactgactc acatagttgt agttgcgctt ttcgttcaag

6721 agagcagtag ggagggccat cttgaagttt ggccaagtaa cagactgcac caacccctgg

6781 gtcaagatag tccatacagc tttgggataa ttgacccctg accatccacc atacgcagca

6841 ttgaatacta cagtatccag cctgggaatg cgtacatttc gtagatattc gccctgcagc

6901 ccctcagggt tgctcaccgg acctcgtaac aaagcatttg cgaaagaata caccccgcgc

6961 aagtcacaca ggtcaagctg caagctcaga acatggattc gcgcaatggt atcttcccaa

7021 cgatatgaac tcccagcccg cgaacgtagt gctgtagaag attgcgcagc cttgttggcg

7081 tagccacgaa gggtttggat cgtttgtaag gacttggact tggatcgtgt ggtagggatg

7141 aggatgaggt gggagcgcaa agagcgtgta gcaaggaatt cgtcgataag tcgttctcca

7201 atactgagac cgataccact tgagttgcat tagctgtcgc tccctatata tagcctggag

7261 cttttgaaca aacctattgg cacctgtgat cagaacaaaa agttgttcct gacccgagac

7321 tccctcccag ggggctgtcg ccggcgacaa ttgggagccc atttggatgc ttgggtagaa

7381 taggtaagtc agattgaatc tgaaataaag ggaggaaggg cgaacttaag aaggtatgac

7441 cgggtcgtcc acttaccttg cttgacaaac gcaccaagtt atcgtgcacc aagcagcaga

7501 tgataataat gtcctcgttc ctgtctgcta ataagagtca cacttcgagc gccgccgcta

7561 ctgctacaag tggggctgat ctgaccagtt gcctaaatga accatcttgt caaacgacac

7621 aaattttgtg ctcaccgcct ggacgactaa accaaaatag gcattcattg ttgacctcca

7681 ctagctccag ccaagcccaa aaaatggcgc gccatatggt acgtaaaacg acggccagtg

7741 agcgcgcgta atacgactca ctatagggcg aattgggtac tcaaattggt tataaatgta

7801 ggtattacct gtacatttta tttattcgag aaaaacaaac aaaccaaaac gatgcagcga

7861 tcactaccgg cgtcatccgg aattgaaacg gcgcgcgttg aggggggcct tcttgttcgg

7921 acacacacgg ggccgccaaa gtggggccca tacggctgcc catcccttgc tctccaatac

7981 cctggtcatg tgactcgaaa ttggagactc acaaagatgc tttgaaaccc cgccgggaac

8041 tttttctcac agacacaatc agtcgacctt atcatcgcaa aatcgacaaa cctcaaaaag

8101 aactcgaaga gtcagtctcc cttgcggtta aactgattga tatctcgtgt tctctaaacc

8161 ttcaataagt gagttactgc gcacaaagcc gactatactg agcaccaacg cgaattgctc

8221 acgttgtcgc ctaacagatc ttggctttcg taggaaccca atcttcaaag cggccgcctc

8281 gagggtac

//

1. ***pTEL-Fen::MoALB1***


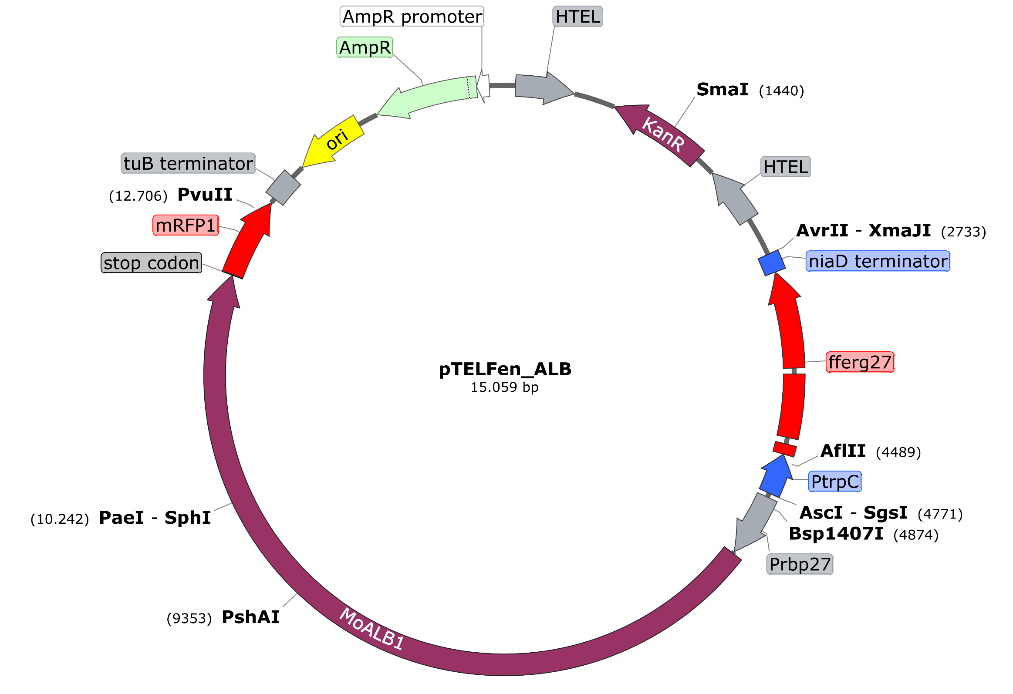


LOCUS pTELFen_ALB 15059 bp DNA circular SYN 20-MAR-2024

DEFINITION synthetic circular DNA

ACCESSION .

VERSION .

KEYWORDS .

SOURCE synthetic DNA construct

ORGANISM synthetic DNA construct

REFERENCE 1 (bases 1 to 15059)

AUTHORS RiBa

TITLE Direct Submission

JOURNAL -

COMMENT -

FEATURES Location/Qualifiers

source 1..15059

/mol_type="other DNA"

/organism="synthetic DNA construct"

misc_feature 93..585

/label=HTEL

CDS complement(931..1746)

/label=KanR

misc_feature complement(1916..2406)

/label=HTEL

misc_feature 2736..2894

/label=niaD terminator

/note="3'"

CDS complement(join(2895..3707,3757..4284,4340..4426))

/codon_start=1

/label=fferg27

/note="fferg27 (FFUJ_04356)"

/translation="MGSQLSPATAPWEGVSGQEQLFVLITGANSGIGLSIGERLIDEFL

ATRSLRSHLILIPTTRSKSKSLQTIQTLRGYANKAAQSSTALRSRAGSSYRWEDTIARI

HVLSLQLDLCDLRGVYSFANALLRGPVSNPEGLQGEYLRNVRIPRLDTVVFNAAYGGWS

GVNYPKAVWTILTQGLVQSVTWPNFKMALPTALLNEKRNYNYPKEPLLGEVFTACVFGH

YILAHELLPLLSRRSETETPGRLVWSSSLEAVDSVLDMSDFQCFNGKGPYESAKRVTDI

LSLTATLPAAMPSSSRFFTPDDPSEARDKPIRPRMYLTHPGIVASTLFPVPWFLMWAYE

LALLISRWIGSPWHNTDSYTGAKSPVWIALQEQSALDELGAERIKWGSSSNRHMQVEVK

KTEVEGWGWEGKVEDAAALEADTAVGVFRKTIGRKRGAKDVTKEDIVRFEELGAECWER

MENMRHEWETILRVKKA"

misc_feature complement(4427..4770)

/label=PtrpC

/note="PtrpC"

misc_feature 4807..5334

/label=Prbp27

CDS 5353..12138

/codon_start=1

/label=MoALB1

/translation="MLTTIKMADKLAFLLFGDQSLDTHGFLAEFYRHEDRGILAKAFLD

QAAQALRKEIERLPRLERSRLPVFRTLQQLNERYHAQITKHSGLDGALLCVAQLAHYIE

YVDSSHDLPTSHSLVACLAACLIPTVQLT*AILLPCSHAEKNHEDVTQHDKTYLVGLCS

GLFAASAIASTPSLSSLVPVAVQTVLMAFRTGAHVAALAERLEPVSERSESWTYIVPGF

TEEAAQKEIAGFHESHVSITSSTSSP*IIS*QVSHHQGIPQASQVYVSASSSANVAISG

PPTTLKAFRAATSSLTKATAIPVYGPYHAPHLHRPEDVDNILGLNNPEMIDTFLNTKPR

SSVMSCLTGTWFNESTTRALFAAVVSEILTQTLRFHNVLDGCLNLARGFDGKSCLIIPF

GPTQHAATLANLLKAETNLDIILRKAPQISKESTDSVIGNHGRGSRDKLAIVGMAGRFP

DAASHEKLWELLSKGLDVHRVVPADRFPVETHYDPNGKVINSSHTPYGCWIEKPGMFDP

RFFNMSPREAFQTDPMQRMALTTAYEALEMSGYVPNRTPSTRLDRIGTFYGQTSDDWRE

INAAQEVDTYYITGGVRAFGPGRINYHLGFSGPSLNVDTACSSSAAAMNVACSSLWARD

CDTAIVGGLSCMTNPDIFAGLSRGQFLSKKGPCATFDNNADGYCRGDSCASVIVKRLDD

AIADHDRVLAVILGTATNHSADAISITHPHGPTQSLLSSAILDDAGVDPLDVDYVEMHG

TGTQAGDGTEMVSVTNVFAPANRKRPADRPLYLGAVKANLGHGEAASGVTALCKVLLML

QKNAIPPHVGIKKGSVINKTFPKDLGERNVNIAFHLTPFKRSDGKPRRVFINNFSAAGG

NTGLLLEDAPPVEPATQDPRNVHVITITGKSKSAMIHNAERLIQFMDDNDDTPISHVAY

STTARRIQHYWRMNVTAQDLGEAKQALKNRLQENFVPVSPEPPKVAFMFTGQGSHYAGL

GKELYANHAVFRQAINEFDRISQIHGFPSFMPLVDGSEPDVAKLEPLIVQLGLCCFEMA

LAKLWASWGVKPAAVMGHSLGEYAALHVAGVLSASDTIYLVGARAKLLVEKCTAGTHAM

LAVMGSVSTVQEALGERADSTNVACINGPRETVLSGEASEMAEIAQQLGAAGFKCTQLK

VPFAFHSAQVDPILDDFEKLARSVRFNTPKVPVISPLLGKLVDDEPINPKYLRDHARDA

VDFLGGLVSAQSAGAIDEKTVWLEVGPHPVCANMVKAAFGVSTVAVPTLRRNEATYKTL

SASLCTLHTAGLNIDWNEYHNDFLSSVRHLDLPSYAFDEKNYWLQYEGDWCLSKNRIAK

ALPAAAAVAKPKLETTTVQKVVREEIKGDVAVVEIESNLSREDLRGVVSGHLVNGAPLC

PSSLYGDMAITTCQYAYKLLRPESENIGVNVAHMEVPKTLIFDDAAESHILRLTVTANA

AQNSADLVFHTGEGAKRVDHANCKVYFGNTEDWQGEFDRVSYLIKSRVDGLIDSEKDGK

ASKIGRGLAYKLFAALVDYTPRYRGMEEVVLDSETCEATAQVKFQTTEADGNYVFSPYW

IDSLCHISGFIINGTDAVDSREQVFISHGWGSLRFSELPVASKKYRSYIRMQPIKGNKV

YAGDVYVFDGEKIIGVCGDLKFQAIPRKVLNMMLPPRGAAAIAAPARAAPAPAAKPAAK

EKKTSSTKEKSKSVTPKNIGKVNQKLASVVTQVLDILAKEVGVSHDELADNIAFTDLGV

DSLMSLTVSGRIREDMDLSIDSHAFVDHPTIGAFKAFLAQFEGPGGAMEDSSSSSSSVS

GDMDSESDVTTPADESDTASVKGEKMDGKATATDTNELQEAIRNTIATEMGMDVEEIIA

APDLASLGMDSLMSLQILGTLREKTGMDVPSDLFVSNPSLKDVERALGIVPAAPKRPAA

QAAPAKSKASKSEKVKPCPPTTSIPPSQVTATIYKPPRPTEILDTMPHRKATSVLLHGS

HKTATRHLFMVPDGSGCATSYTEMCNVGPNYAVWGLFSPFMKTPEEYNCGVYGMAAKFI

EEMKRRQPVGPYSLAGWSAGGVIAFEIVNQLIKAGDEVENMIIIDAPCPVTIEPLPKSL

HAWFASIGLLGDGDADKIPSWLLPHFAASVTALSNYTAEPIPKDKCPKVMAIWCEDGVC

HLPTDPRPDPYPSGHALFLLDNRVDFGPNRWDEYLDGSKMTMRHMPGNHFSMMHGAMVS

RTSTLPSMPCTSDANHTLTQQAKQLQGFMQEALQ"

misc_feature 12139..12141

/label=stop codon

CDS 12146..12820

/codon_start=1

/product="monomeric derivative of DsRed (Campbell et al.,

2002)"

/label=mRFP1

/translation="ASSEDVIKEFMRFKVRMEGSVNGHEFEIEGEGEGRPYEGTQTAKL

KVTKGGPLPFAWDILSPQFQYGSKAYVKHPADIPDYLKLSFPEGFKWERVMNFEDGGVV

TVTQDSSLQDGEFIYKVKLRGTNFPSDGPVMQKKTMGWEASTERMYPEDGALKGEIKMR

LKLKDGGHYDAEVKTTYMAKKPVQLPGAYKTDIKLDITSHNEDYTIVEQYERAEGRHST

GA"

misc_feature 12863..13096

/label=tuB terminator

rep_origin complement(13210..13798)

/direction=LEFT

/label=ori

/note="high-copy-number ColE1/pMB1/pBR322/pUC origin of

replication"

CDS complement(13969..14829)

/codon_start=1

/gene="bla"

/product="beta-lactamase"

/label=AmpR

/note="confers resistance to ampicillin, carbenicillin, and

related antibiotics"

/translation="MSIQHFRVALIPFFAAFCLPVFAHPETLVKVKDAEDQLGARVGYI

ELDLNSGKILESFRPEERFPMMSTFKVLLCGAVLSRVDAGQEQLGRRIHYSQNDLVEYS

PVTEKHLTDGMTVRELCSAAITMSDNTAANLLLTTIGGPKELTAFLHNMGDHVTRLDRW

EPELNEAIPNDERDTTMPAAMATTLRKLLTGELLTLASRQQLIDWMEADKVAGPLLRSA

LPAGWFIADKSGAGERGSRGIIAALGPDGKPSRIVVIYTTGSQATMDERNRQIAEIGAS

LIKHW"

promoter complement(14830..14934)

/gene="bla"

/label=AmpR promoter

ORIGIN

1 gatctaggcc tgcaggatgc tagcttcaga cgtgtctagg gataacaggg taattcgaac

61 ccccgcgccg cctttgcgag ggtggagttg ccttagggtt agggttaggg ttagggttag

121 ggttagggtt agggttaggg ttagggttag ggttagggtt tagggttagg gttagggtta

181 gggttagggt tagggttagg gtcagggtca ggggtagggt caggggtagg gtcaggggta

241 ggggtagggg tagggtcagg gttagggtta gggttagggt tagggttagg gttagggtta

301 gggtcagggt tagggttagg gttagggagg ggtaggggta ggggtagggt tagggttagg

361 gttagggtta gggttagggt tagggttagg gtcagggtca gggtcagggg taggggtagg

421 ggtaggggta ggggtagggt taggggtagg gttaggggta ggggtagggg tagggttagg

481 gttagggttt agggttaggg ttagggttag ggttagggtt aggggttagg gttagggtta

541 gggttagaag gttagggtta gggttagggt taagggttaa gggttaggtg tggggtgtgg

601 cgaattcctc gacctgcaag cggccgcttg cagggggggg ggggcgctga ggtctgcctc

661 gtgaagaagg tgttgctgac tcataccagg cctgaatcgc cccatcatcc agccagaaag

721 tgagggagcc acggttgatg agagctttgt tgtaggtgga ccagttggtg attttgaact

781 tttgctttgc cacggaacgg tctgcgttgt cgggaagatg cgtgatctga tccttcaact

841 cagcaaaagt tcgatttatt caacaaagcc gccgtcccgt caagtcagcg taatgctctg

901 ccagtgttac aaccaattaa ccaattctga ttagaaaaac tcatcgagca tcaaatgaaa

961 ctgcaattta ttcatatcag gattatcaat accatatttt tgaaaaagcc gtttctgtaa

1021 tgaaggagaa aactcaccga ggcagttcca taggatggca agatcctggt atcggtctgc

1081 gattccgact cgtccaacat caatacaacc tattaatttc ccctcgtcaa aaataaggtt

1141 atcaagtgag aaatcaccat gagtgacgac tgaatccggt gagaatggca aaagcttatg

1201 catttctttc cagacttgtt caacaggcca gccattacgc tcgtcatcaa aatcactcgc

1261 atcaaccaaa ccgttattca ttcgtgattg cgcctgagcg agacgaaata cgcgatcgct

1321 gttaaaagga caattacaaa caggaatcga atgcaaccgg cgcaggaaca ctgccagcgc

1381 atcaacaata ttttcacctg aatcaggata ttcttctaat acctggaatg ctgttttccc

1441 ggggatcgca gtggtgagta accatgcatc atcaggagta cggataaaat gcttgatggt

1501 cggaagaggc ataaattccg tcagccagtt tagtctgacc atctcatctg taacatcatt

1561 ggcaacgcta cctttgccat gtttcagaaa caactctggc gcatcgggct tcccatacaa

1621 tcgatagatt gtcgcacctg attgcccgac attatcgcga gcccatttat acccatataa

1681 atcagcatcc atgttggaat ttaatcgcgg cctcgagcaa gacgtttccc gttgaatatg

1741 gctcataaca ccccttgtat tactgtttat gtaagcagac agttttattg ttcatgatga

1801 tatattttta tcttgtgcaa tgtaacatca gagattttga gacacaacgt ggctttcccc

1861 cccccccctg caagcggccg cttgcaggtc gaggaattcg ccacacccca cacctaaccc

1921 ttaaccctta accctaaccc taaccctaac cctaacccta accctaaccc taacccctaa

1981 ccctaaccct aaccctaacc ctaaccctaa accctaaccc taaccctacc cctaccccta

2041 cccctaaccc tacccctaac cctaccccta cccctacccc tacccctacc cctgaccctg

2101 accctgaccc taaccctaac cctaacccta accctaaccc taaccctaac cctaccccta

2161 cccctacccc tccctaaccc taaccctaac cctgacccta accctaaccc taaccctaac

2221 cctaacccta accctaaccc tgaccctacc cctaccccta cccctgaccc tacccctgac

2281 cctacccctg accctgaccc taaccctaac cctaacccta accctaaccc taaccctaaa

2341 ccctaaccct aaccctaacc ctaaccctaa ccctaaccct aaccctaacc ctaaccctaa

2401 ccctaaggca actccaccct cgcaaaggcg gcgcgggggt tcgaattacc ctgttatccc

2461 tagatacgtc tgctttttgt tgacttccat tgttcattcc acggacaaaa acagagaaag

2521 gaaacgacag aggccaaaaa gctcgctttc agcacctgtc gtttcctttc ttttcagagg

2581 gtattttaaa taaaaacatt aagttatgac gaagaagaac ggaaacgcct taaaccggaa

2641 aattttcata aatagcgaaa acccgcgagg tcgccgcccc gtaacaaggc ggatcgccgg

2701 aaaggacccg caaatgataa taattatcaa ttcctaggct cactgataca tctggcacct

2761 actttataat aatactttat tgaaatactt tatagatact atattcaaac atcctctctc

2821 ccattatttt agacgcagtt caatggcaaa aactacaacc atatctaaac cacctctcag

2881 ttacttaaaa cctcctatgc ctttttaacc ctaagaatgg tttcccactc gtgtcgcatg

2941 ttctccatcc tttcccagca ttcggcgccc agttcctcga atctcacgat atcttccttg

3001 gtcacgtctt tagcgcctct ctttcgcccg atggtcttcc tgaaaacacc aacagcggtg

3061 tccgcttcaa gcgccgccgc atcctcgact ttgccctccc aaccccagcc ttcaacttct

3121 gtcttcttaa cttcgacctg catgtgacgg ttcgagctgc tgccccactt gatgcgctct

3181 gcacctagtt catcaagcgc agattgctct tggagcgcaa tccaaacagg ggatttggca

3241 ccggtatagc tgtctgtgtt gtgccagggc gaaccaatcc agcgactgat cagaagagca

3301 agttcatatg cccacatcag aaaccaggga actgggaata gggtactggc aacaataccc

3361 ggatgggtga gatacatgcg cggtcgaatg ggtttatcgc gggcctcgct ggggtcgtcg

3421 ggggtgaaga agcggctgga agatggcatg gctgcgggga gagttgcagt gagggagaga

3481 atgtcggtga ctcgtttagc ggattcgtac gggcctttac cattgaagca ctggaagtcg

3541 gacatatcaa gaacactgtc gacggcctca agactgctgg accagacaag acggccgggt

3601 gtttcggttt cggatcgccg actaagtagt ggaagaagtt catgggccaa tatataatga

3661 ccaaaaacac aggctgtaaa cacctcgccg agtaaaggtt ccttgggctg cattcagtca

3721 atgggtgaaa tcattgagtt actgcgactg actcacatag ttgtagttgc gcttttcgtt

3781 caagagagca gtagggaggg ccatcttgaa gtttggccaa gtaacagact gcaccaaccc

3841 ctgggtcaag atagtccata cagctttggg ataattgacc cctgaccatc caccatacgc

3901 agcattgaat actacagtat ccagcctggg aatgcgtaca tttcgtagat attcgccctg

3961 cagcccctca gggttgctca ccggacctcg taacaaagca tttgcgaaag aatacacccc

4021 gcgcaagtca cacaggtcaa gctgcaagct cagaacatgg attcgcgcaa tggtatcttc

4081 ccaacgatat gaactcccag cccgcgaacg tagtgctgta gaagattgcg cagccttgtt

4141 ggcgtagcca cgaagggttt ggatcgtttg taaggacttg gacttggatc gtgtggtagg

4201 gatgaggatg aggtgggagc gcaaagagcg tgtagcaagg aattcgtcga taagtcgttc

4261 tccaatactg agaccgatac cacttgagtt gcattagctg tcgctcccta tatatagcct

4321 ggagcttttg aacaaaccta ttggcacctg tgatcagaac aaaaagttgt tcctgacccg

4381 agactccctc ccagggggct gtcgccggcg acaattggga gcccatttgg atgcttgggt

4441 agaataggta agtcagattg aatctgaaat aaagggagga agggcgaact taagaaggta

4501 tgaccgggtc gtccacttac cttgcttgac aaacgcacca agttatcgtg caccaagcag

4561 cagatgataa taatgtcctc gttcctgtct gctaataaga gtcacacttc gagcgccgcc

4621 gctactgcta caagtggggc tgatctgacc agttgcctaa atgaaccatc ttgtcaaacg

4681 acacaaattt tgtgctcacc gcctggacga ctaaaccaaa ataggcattc attgttgacc

4741 tccactagct ccagccaagc ccaaaaaatg gcgcgccata tggtacgtaa aacgacggcc

4801 agtgagcgcg cgtaatacga ctcactatag ggcgaattgg gtactcaaat tggttataaa

4861 tgtaggtatt acctgtacat tttatttatt cgagaaaaac aaacaaacca aaacgatgca

4921 gcgatcacta ccggcgtcat ccggaattga aacggcgcgc gttgaggggg gccttcttgt

4981 tcggacacac acggggccgc caaagtgggg cccatacggc tgcccatccc ttgctctcca

5041 ataccctggt catgtgactc gaaattggag actcacaaag atgctttgaa accccgccgg

5101 gaactttttc tcacagacac aatcagtcga ccttatcatc gcaaaatcga caaacctcaa

5161 aaagaactcg aagagtcagt ctcccttgcg gttaaactga ttgatatctc gtgttctcta

5221 aaccttcaat aagtgagtta ctgcgcacaa agccgactat actgagcacc aacgcgaatt

5281 gctcacgttg tcgcctaaca gatcttggct ttcgtaggaa cccaatcttc aaagcggccg

5341 cctcgagggt acatgctgac aaccatcaaa atggcggaca agctggcttt cctcctcttc

5401 ggagaccaat ctctggacac tcatggattc ctcgccgagt tctaccgtca cgaggatcgg

5461 ggaatactcg ccaaggcctt tctcgaccag gccgcccaag ctctgaggaa agagatcgaa

5521 aggctaccaa ggctggagag gtcgaggctg cccgtgttcc gcacactgca acaactaaac

5581 gagcggtatc atgctcaaat caccaagcac tccggcctcg acggtgctct tctatgtgtt

5641 gcccagctcg ctcactacat tgagtatgtt gattcatcac acgatctacc tacctcccac

5701 agcctcgtcg cttgcttggc tgcttgcttg attccaacag tacaactaac ctgagcaatt

5761 ctcctccctt gtagccacgc agagaagaac catgaagatg tcacacagca tgacaaaacc

5821 taccttgtcg gtctctgctc aggcctcttc gctgcgtccg caatcgcttc aaccccatca

5881 ttgtcttctc ttgttccggt cgccgtgcaa acagttctca tggctttccg gaccggtgca

5941 cacgttgccg ccctcgccga aaggctggag ccggtcagcg agcgctccga gagctggact

6001 tacattgttc ccggcttcac tgaggaggcc gcacaaaagg aaatcgctgg attccacgag

6061 tcacatgtaa gcataacctc ctcgaccagc tcaccctaga tcatcagcta acaggtctcc

6121 caccatcagg gtatccccca agcgagccag gtgtacgtaa gcgccagctc gtccgctaat

6181 gtcgccatct ctggccctcc caccacactc aaggctttca gggccgccac aagctccctc

6241 accaaggcca cggccatccc ggtctacggc ccgtaccacg ccccccacct ccaccgccct

6301 gaggatgtcg acaacatcct gggcctcaac aaccccgaga tgattgacac cttcctcaac

6361 accaagcccc gctcctccgt catgtcctgc ctaactggca cctggttcaa cgagtccacc

6421 acccgcgctc tcttcgccgc tgtcgtctcc gagattctca cccagaccct gaggtttcac

6481 aacgtgctcg acggctgctt aaatctggct cgtggttttg acggaaagtc gtgcctcatc

6541 atccccttcg gccccacaca gcacgccgcc actctcgcca acctcctcaa ggctgagacc

6601 aacctggaca tcatcctccg caaggccccg caaatatcca aggagagcac cgactcggtc

6661 attggaaacc atggccgtgg cagccgtgac aagctggcca ttgtcggtat ggccggtcgt

6721 ttccccgatg ctgccagcca cgagaagctg tgggagcttc tttccaaggg actagacgtt

6781 caccgcgtcg tccctgccga tcgtttcccg gtcgagactc actacgaccc taacggcaag

6841 gttatcaact ccagtcacac cccttacggc tgctggattg agaagcctgg catgttcgac

6901 cctcgtttct tcaacatgtc accgcgtgag gctttccaga ccgaccccat gcagaggatg

6961 gccctgacaa ccgcctacga ggctctcgag atgagcggtt acgtgcccaa caggacaccc

7021 tcgaccaggc tggaccgtat cggtaccttc tacggtcaga cttccgatga ctggcgtgag

7081 atcaacgcgg cccaggaggt cgacacctac tacatcacgg gaggtgtccg agccttcggc

7141 cccggtagaa tcaactacca ccttggcttc agcggtcctt cgctgaacgt ggacactgcc

7201 tgctcttcca gtgccgctgc catgaacgtt gcctgctctt cgctgtgggc ccgtgactgt

7261 gacaccgcca ttgttggtgg tctgtcctgc atgaccaacc ccgacatttt cgcgggtctg

7321 tctcgtggac agttcctttc caagaagggc ccctgtgcca ccttcgacaa caatgccgat

7381 ggatactgcc gtggtgacag ttgcgcctca gtcattgtga agaggctcga cgatgccatt

7441 gccgaccatg atcgcgttct cgctgtcatc ctgggcactg ccaccaacca ctcggctgat

7501 gccatctcca tcacccaccc tcacggtcct acgcagtcgc ttctgtcgtc tgctatcctg

7561 gacgatgctg gtgttgaccc tctcgatgtt gactacgtcg agatgcacgg taccggtact

7621 caggccggtg acggaacaga gatggtctca gtcaccaacg tctttgctcc tgccaacagg

7681 aagagacctg ctgacagacc cctgtacctt ggtgccgtca aggccaacct gggtcacggt

7741 gaggccgcat cgggtgtcac ggctctgtgc aaggttcttc tcatgctcca gaagaacgcc

7801 attcctcccc acgtcggtat caagaaggga tccgtcatca acaagacctt ccccaaggac

7861 cttggtgagc gcaacgtcaa cattgctttc cacttgaccc ccttcaagag gtcggatggc

7921 aagccccgca gggttttcat caacaacttc agcgctgctg gtggtaacac cggtctgctg

7981 cttgaggatg ctcctcccgt tgagcctgcg actcaggacc cccgcaacgt ccacgtgatc

8041 accatcactg gcaagtccaa gtcggccatg atccacaacg ccgagaggct catccagttc

8101 atggatgaca acgatgacac tcccatctct cacgttgcct attccaccac cgctcgtcgc

8161 atccagcact actggcgcat gaacgtgact gctcaggatc tgggtgaggc taagcaggcc

8221 ctcaagaacc gcctgcagga gaactttgtt cccgtctccc ccgagcctcc caaggttgcc

8281 ttcatgttca ctggccaggg ctctcactac gctggtcttg gaaaggagct gtacgccaac

8341 cacgctgtct tccgccaggc catcaacgag ttcgacagga tctcgcagat ccacggcttc

8401 ccgagcttca tgcctctggt cgacggcagc gagcctgatg tcgccaagct cgagcccctc

8461 atcgtccagc ttggtctctg ctgcttcgag atggccttgg ccaagctctg ggcttcttgg

8521 ggtgtcaagc ctgctgcggt catgggccac agcctgggcg agtacgctgc tctccacgtt

8581 gccggtgttc tgtctgccag cgacaccatc tacctcgttg gtgctcgtgc caagcttctc

8641 gttgagaagt gcactgccgg cacccacgcc atgttggctg tcatgggatc cgtctccact

8701 gtccaggagg ctcttggaga gcgcgccgac tctaccaacg ttgcttgcat caacggaccc

8761 cgcgagacgg ttctcagtgg tgaggcttct gagatggctg agatcgctca gcagcttggt

8821 gccgctggct tcaagtgcac tcagctcaag gtgcccttcg ccttccactc ggcccaggtc

8881 gaccccatcc ttgatgactt tgagaagctg gcccgctctg tccgcttcaa cacccccaag

8941 gtccccgtca tctcgcccct ccttggcaag ctcgttgacg atgagcccat caaccccaag

9001 tacctgcgcg atcatgctcg tgatgctgtt gacttccttg gcgggcttgt ttctgcccag

9061 tcagccggcg ccatcgatga gaagactgtc tggctcgagg tcggccccca ccccgtctgc

9121 gccaacatgg tcaaggctgc tttcggcgtc tcgaccgtcg ctgtccccac tcttcgccgc

9181 aacgaggcca cttacaagac cctgagcgcc agtctctgca ctctccacac tgctggtctg

9241 aacattgact ggaacgagta ccacaacgac ttcctctctt ccgtcaggca cttggatctc

9301 ccctcgtatg ctttcgacga gaagaactac tggctccagt acgagggaga ctggtgtctc

9361 agcaagaacc gcatcgccaa ggccctgcct gctgctgctg ccgtcgccaa gcccaagctc

9421 gagacgacca ccgtgcagaa ggttgtccgc gaggagatca agggtgatgt tgccgtcgtc

9481 gagatcgagt ccaacctgtc ccgcgaggac cttcgtggcg tcgtttccgg tcacttggtc

9541 aacggtgctc ctctctgccc ttcttcgctg tatggagaca tggccatcac tacttgtcaa

9601 tatgcctaca agctgctccg tcccgagtct gagaacattg gtgtcaacgt cgctcacatg

9661 gaggttccca agaccctcat cttcgacgac gctgctgaga gccacatcct gaggctcaca

9721 gtcactgcca atgctgccca gaacagcgct gacctggtct tccacactgg cgagggtgcc

9781 aagcgtgttg accacgccaa ctgcaaggtc tactttggca acactgagga ctggcagggc

9841 gagttcgacc gcgtctctta cctcatcaag tctcgcgttg acggtctcat tgactctgag

9901 aaggatggca aggcatccaa gatcggccgt ggcttggctt acaagctgtt cgccgcactg

9961 gtcgactaca ctccccgcta ccgcggtatg gaggaggtcg tcctcgacag cgagacttgc

10021 gaggctactg ctcaggtcaa gttccagacc accgaggccg atggcaacta cgtcttcagc

10081 ccctactgga tcgacagtct gtgccacatc tcgggcttca tcatcaacgg aaccgacgct

10141 gtcgactcgc gcgagcaggt cttcatctcc cacggatggg gctcgctcag gttctctgag

10201 ctccctgttg cctccaagaa gtaccgcagc tacatccgca tgcagcccat caagggtaac

10261 aaggtctacg ctggtgacgt ctacgtcttt gacggcgaga agatcatcgg cgtgtgcggt

10321 gacctgaagt tccaggccat cccccgcaag gtcctcaaca tgatgctgcc cccgcgtgga

10381 gctgctgcca ttgccgcccc cgcccgcgct gctcctgctc ccgctgccaa gccggctgcc

10441 aaggagaaga agacctcctc caccaaggag aagagcaaga gcgtgacccc caagaacatc

10501 ggcaaggtca accagaagct cgcctcggtc gtcacgcagg tgctcgacat cctcgccaag

10561 gaggttggtg tcagccacga cgagcttgct gacaacattg ccttcaccga tctgggtgtc

10621 gattcgctga tgtcgcttac tgtcagtggt cgcattcgtg aggacatgga cctgagcatc

10681 gactcgcacg cctttgtcga ccaccctacc attggtgctt tcaaggcctt ccttgctcag

10741 ttcgagggcc ccggcggcgc catggaggac agctccagca gcagctcttc cgtctcgggt

10801 gacatggact ccgagtcgga cgtcaccacc cctgctgacg agagcgatac tgcctcggtc

10861 aagggtgaga agatggacgg caaggctact gctactgaca ccaacgagct ccaggaggct

10921 atccgcaaca ccattgccac cgagatgggc atggacgttg aggagatcat cgccgccccc

10981 gacttggcca gcttgggtat ggactcgctc atgagtcttc agatcctggg caccctcagg

11041 gagaagactg gcatggatgt cccctcggac ctctttgtct ccaacccttc gctgaaggac

11101 gtcgagcgtg ctctgggcat cgtcccggct gccccaaagc gccctgccgc tcaggctgcc

11161 cccgccaagt ccaaggcttc caagtctgag aaggtcaagc cttgcccacc caccacctcg

11221 atccccccat ctcaggtgac ggccaccatc tacaagccac cccgccccac cgagatcctc

11281 gacacaatgc ctcaccgcaa ggccacatcg gtgctgctgc atggtagcca caagaccgcc

11341 acccgccacc tcttcatggt acccgacgga agcggctgcg ccacctcgta caccgagatg

11401 tgcaatgtcg gtcccaacta cgccgtctgg ggtctcttct cgcccttcat gaagaccccc

11461 gaggagtaca actgcggtgt ctacggcatg gcggccaagt tcatcgagga gatgaagcgc

11521 cgccagcccg tcggacccta cagccttgct ggctggtccg ccggtggtgt catcgccttt

11581 gagattgtca atcagctcat caaggccggc gacgaggttg agaacatgat catcatcgac

11641 gctccttgcc ccgtgaccat tgagcccctc cccaagtccc tgcacgcctg gttcgcatcc

11701 atcggcctgc tgggcgacgg cgacgcggac aagatcccct cgtggctcct gccccacttt

11761 gccgcttcgg tcacggccct ctccaactac acagccgagc cgatccccaa ggacaagtgc

11821 cccaaggtca tggccatctg gtgcgaggat ggtgtctgcc accttcccac cgaccccagg

11881 cctgaccctt acccctcggg acacgccctc ttcctcctcg acaacagggt cgactttggc

11941 cccaaccgct gggacgagta cctcgatggc agcaagatga ccatgaggca catgcccggt

12001 aaccactttt cgatgatgca cggcgctatg gtaagtcgca cctcgacact cccatccatg

12061 ccttgtactt cagacgctaa ccataccctc actcaacagg ccaagcagct tcaaggtttc

12121 atgcaagagg ctctgcaata atgctgcctc ctccgaggac gtcatcaagg agttcatgcg

12181 cttcaaggtg cgcatggagg gctccgtgaa cggccacgag ttcgagatcg agggcgaggg

12241 cgagggccgc ccctacgagg gcacccagac cgccaagctg aaggtgacca agggcggccc

12301 cctgcccttc gcctgggaca tcctgtcccc tcagttccag tacggctcca aggcctacgt

12361 gaagcacccc gccgacatcc ccgactactt gaagctgtcc ttccccgagg gcttcaagtg

12421 ggagcgcgtg atgaacttcg aggacggcgg cgtggtgacc gtgacccagg actcctccct

12481 gcaggacggc gagttcatct acaaggtgaa gctgcgcggc accaacttcc cctccgacgg

12541 ccccgtaatg cagaagaaga caatgggctg ggaggcctcc accgagcgga tgtaccccga

12601 ggacggcgcc ctgaagggcg agatcaagat gaggctgaag ctgaaggacg gcggccacta

12661 cgacgccgag gtcaagacca cctacatggc caagaagccc gtgcagctgc ccggcgccta

12721 caagaccgac atcaagctgg acatcacctc ccacaacgag gactacacca tcgtggaaca

12781 gtacgagcgc gccgagggcc gccacagtac tggagcatga atcattccac tcaacattca

12841 ggctcctctg cgcacgtaaa gtgccaaagg caataccctg ctcggtggaa tgccgccggg

12901 cttgtcgatt ttacgcacat atgcgcattc ttgacttgaa gcggaggagt tcttcgttgc

12961 gggttacagt gttttaataa aagaatggtc aaatcaaact gctagatata cctgtcagac

13021 actctagttg ttgaccccta tactcttaat acatcagaca gtacatgcat gttgcatgat

13081 gatgatatgt ctgtttagat tccagtgtct actgctggcc tagtttctcg gtactatgca

13141 tactacatgc atgtgagcaa aaggccagca aaaggccagg aaccgtaaaa aggccgcgtt

13201 gctggcgttt ttccataggc tccgcccccc tgacgagcat cacaaaaatc gacgctcaag

13261 tcagaggtgg cgaaacccga caggactata aagataccag gcgtttcccc ctggaagctc

13321 cctcgtgcgc tctcctgttc cgaccctgcc gcttaccgga tacctgtccg cctttctccc

13381 ttcgggaagc gtggcgcttt ctcatagctc acgctgtagg tatctcagtt cggtgtaggt

13441 cgttcgctcc aagctgggct gtgtgcacga accccccgtt cagcccgacc gctgcgcctt

13501 atccggtaac tatcgtcttg agtccaaccc ggtaagacac gacttatcgc cactggcagc

13561 agccactggt aacaggatta gcagagcgag gtatgtaggc ggtgctacag agttcttgaa

13621 gtggtggcct aactacggct acactagaag gacagtattt ggtatctgcg ctctgctgaa

13681 gccagttacc ttcggaaaaa gagttggtag ctcttgatcc ggcaaacaaa ccaccgctgg

13741 tagcggtggt ttttttgttt gcaagcagca gattacgcgc agaaaaaaag gatctcaaga

13801 agatcctttg atcttttcta cggggtctga cgctcagtgg aacgaaaact cacgttaagg

13861 gattttggtc atgagattat caaaaaggat cttcacctag atccttttaa attaaaaatg

13921 aagttttaaa tcaatctaaa gtatatatga gtaaacttgg tctgacagtt accaatgctt

13981 aatcagtgag gcacctatct cagcgatctg tctatttcgt tcatccatag ttgcctgact

14041 ccccgtcgtg tagataacta cgatacggga gggcttacca tctggcccca gtgctgcaat

14101 gataccgcga gacccacgct caccggctcc agatttatca gcaataaacc agccagccgg

14161 aagggccgag cgcagaagtg gtcctgcaac tttatccgcc tccatccagt ctattaattg

14221 ttgccgggaa gctagagtaa gtagttcgcc agttaatagt ttgcgcaacg ttgttgccat

14281 tgctgcaggc atcgtggtgt cacgctcgtc gtttggtatg gcttcattca gctccggttc

14341 ccaacgatca aggcgagtta catgatcccc catgttgtgc aaaaaagcgg ttagctcctt

14401 cggtcctccg atcgttgtca gaagtaagtt ggccgcagtg ttatcactca tggttatggc

14461 agcactgcat aattctctta ctgtcatgcc atccgtaaga tgcttttctg tgactggtga

14521 gtactcaacc aagtcattct gagaatagtg tatgcggcga ccgagttgct cttgcccggc

14581 gtcaacacgg gataataccg cgccacatag cagaacttta aaagtgctca tcattggaaa

14641 acgttcttcg gggcgaaaac tctcaaggat cttaccgctg ttgagatcca gttcgatgta

14701 acccactcgt gcacccaact gatcttcagc atcttttact ttcaccagcg tttctgggtg

14761 agcaaaaaca ggaaggcaaa atgccgcaaa aaagggaata agggcgacac ggaaatgttg

14821 aatactcata ctcttccttt ttcaatatta ttgaagcatt tatcagggtt attgtctcat

14881 gagcggatac atatttgaat gtatttagaa aaataaacaa ataggggttc cgcgcacatt

14941 tccccgaaaa gtgccacctg acgtctaaga aaccattatt atcatgacat taacctataa

15001 aaataggcgt atcacgaggc cctttcgtct tcaagaattc gcggccccgc atgggccca

//

1. ***pTEL-Fen::MoPKC***


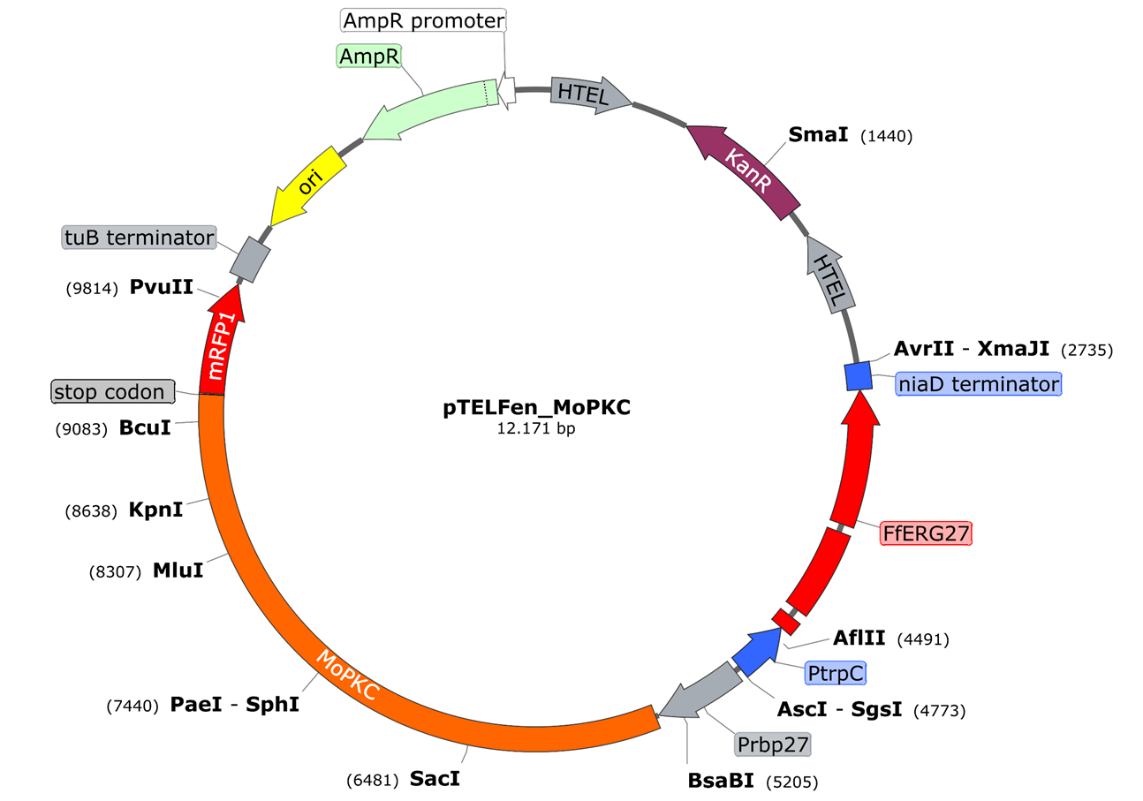


LOCUS pTELFen_MoPKC 12171 bp DNA circular SYN 14-MAR-2024

DEFINITION synthetic circular DNA

ACCESSION .

VERSION .

KEYWORDS .

SOURCE synthetic DNA construct

ORGANISM synthetic DNA construct

REFERENCE 1 (bases 1 to 12171)

AUTHORS RiBa

TITLE Direct Submission

JOURNAL -

COMMENT VNTDATE|927936000|

-

FEATURES Location/Qualifiers

source 1..12171

/mol_type="other DNA"

/organism="synthetic DNA construct"

misc_feature 93..585

/label=HTEL

CDS complement(931..1746)

/codon_start=1

/product="aminoglycoside phosphotransferase"

/label=KanR

/note="confers resistance to kanamycin in bacteria or G418

(Geneticin(R)) in eukaryotes"

/translation="MSHIQRETSCSRPRLNSNMDADLYGYKWARDNVGQSGATIYRLYG

KPDAPELFLKHGKGSVANDVTDEMVRLNWLTEFMPLPTIKHFIRTPDDAWLLTTAIPGK

TAFQVLEEYPDSGENIVDALAVFLRRLHSIPVCNCPFNSDRVFRLAQAQSRMNNGLVDA

SDFDDERNGWPVEQVWKEMHKLLPFSPDSVVTHGDFSLDNLIFDEGKLIGCIDVGRVGI

ADRYQDLAILWNCLGEFSPSLQKRLFQKYGIDNPDMNKLQFHLMLDEFF"

misc_feature complement(1916..2408)

/label=HTEL

misc_feature 2738..2896

/label=niaD terminator

CDS complement(join(2897..3709,3759..4286,4342..4428))

/codon_start=1

/label=FfERG27

/translation="MGSQLSPATAPWEGVSGQEQLFVLITGANSGIGLSIGERLIDEFL

ATRSLRSHLILIPTTRSKSKSLQTIQTLRGYANKAAQSSTALRSRAGSSYRWEDTIARI

HVLSLQLDLCDLRGVYSFANALLRGPVSNPEGLQGEYLRNVRIPRLDTVVFNAAYGGWS

GVNYPKAVWTILTQGLVQSVTWPNFKMALPTALLNEKRNYNYPKEPLLGEVFTACVFGH

YILAHELLPLLSRRSETETPGRLVWSSSLEAVDSVLDMSDFQCFNGKGPYESAKRVTDI

LSLTATLPAAMPSSSRFFTPDDPSEARDKPIRPRMYLTHPGIVASTLFPVPWFLMWAYE

LALLISRWIGSPWHNTDSYTGAKSPVWIALQEQSALDELGAERIKWGSSSNRHMQVEVK

KTEVEGWGWEGKVEDAAALEADTAVGVFRKTIGRKRGAKDVTKEDIVRFEELGAECWER

MENMRHEWETILRVKKA"

misc_feature complement(4429..4772)

/label=PtrpC

misc_feature 4809..5336

/label=Prbp27

misc_feature 5355..9246

/label=MoPKC

misc_feature 9247..9249

CDS 9254..9928

/codon_start=1

/product="monomeric derivative of DsRed (Campbell et al.,

2002)"

/label=mRFP1

/translation="ASSEDVIKEFMRFKVRMEGSVNGHEFEIEGEGEGRPYEGTQTAKL

KVTKGGPLPFAWDILSPQFQYGSKAYVKHPADIPDYLKLSFPEGFKWERVMNFEDGGVV

TVTQDSSLQDGEFIYKVKLRGTNFPSDGPVMQKKTMGWEASTERMYPEDGALKGEIKMR

LKLKDGGHYDAEVKTTYMAKKPVQLPGAYKTDIKLDITSHNEDYTIVEQYERAEGRHST

GA"

misc_feature 9971..10204

/label=tuB terminator

rep_origin complement(10322..10910)

/direction=LEFT

/label=ori

/note="high-copy-number ColE1/pMB1/pBR322/pUC origin of

replication"

CDS complement(11081..11941)

/codon_start=1

/gene="bla"

/product="beta-lactamase"

/label=AmpR

/note="confers resistance to ampicillin, carbenicillin, and

related antibiotics"

/translation="MSIQHFRVALIPFFAAFCLPVFAHPETLVKVKDAEDQLGARVGYI

ELDLNSGKILESFRPEERFPMMSTFKVLLCGAVLSRVDAGQEQLGRRIHYSQNDLVEYS

PVTEKHLTDGMTVRELCSAAITMSDNTAANLLLTTIGGPKELTAFLHNMGDHVTRLDRW

EPELNEAIPNDERDTTMPAAMATTLRKLLTGELLTLASRQQLIDWMEADKVAGPLLRSA

LPAGWFIADKSGAGERGSRGIIAALGPDGKPSRIVVIYTTGSQATMDERNRQIAEIGAS

LIKHW"

promoter complement(11942..12046)

/gene="bla"

/label=AmpR promoter

ORIGIN

1 gatctaggcc tgcaggatgc tagcttcaga cgtgtctagg gataacaggg taattcgaac

61 ccccgcgccg cctttgcgag ggtggagttg ccttagggtt agggttaggg ttagggttag

121 ggttagggtt agggttaggg ttagggttag ggttagggtt tagggttagg gttagggtta

181 gggttagggt tagggttagg gtcagggtca ggggtagggt caggggtagg gtcaggggta

241 ggggtagggg tagggtcagg gttagggtta gggttagggt tagggttagg gttagggtta

301 gggtcagggt tagggttagg gttagggagg ggtaggggta ggggtagggt tagggttagg

361 gttagggtta gggttagggt tagggttagg gtcagggtca gggtcagggg taggggtagg

421 ggtaggggta ggggtagggt taggggtagg gttaggggta ggggtagggg tagggttagg

481 gttagggttt agggttaggg ttagggttag ggttagggtt aggggttagg gttagggtta

541 gggttagaag gttagggtta gggttagggt taagggttaa gggttaggtg tggggtgtgg

601 cgaattcctc gacctgcaag cggccgcttg cagggggggg ggggcgctga ggtctgcctc

661 gtgaagaagg tgttgctgac tcataccagg cctgaatcgc cccatcatcc agccagaaag

721 tgagggagcc acggttgatg agagctttgt tgtaggtgga ccagttggtg attttgaact

781 tttgctttgc cacggaacgg tctgcgttgt cgggaagatg cgtgatctga tccttcaact

841 cagcaaaagt tcgatttatt caacaaagcc gccgtcccgt caagtcagcg taatgctctg

901 ccagtgttac aaccaattaa ccaattctga ttagaaaaac tcatcgagca tcaaatgaaa

961 ctgcaattta ttcatatcag gattatcaat accatatttt tgaaaaagcc gtttctgtaa

1021 tgaaggagaa aactcaccga ggcagttcca taggatggca agatcctggt atcggtctgc

1081 gattccgact cgtccaacat caatacaacc tattaatttc ccctcgtcaa aaataaggtt

1141 atcaagtgag aaatcaccat gagtgacgac tgaatccggt gagaatggca aaagcttatg

1201 catttctttc cagacttgtt caacaggcca gccattacgc tcgtcatcaa aatcactcgc

1261 atcaaccaaa ccgttattca ttcgtgattg cgcctgagcg agacgaaata cgcgatcgct

1321 gttaaaagga caattacaaa caggaatcga atgcaaccgg cgcaggaaca ctgccagcgc

1381 atcaacaata ttttcacctg aatcaggata ttcttctaat acctggaatg ctgttttccc

1441 ggggatcgca gtggtgagta accatgcatc atcaggagta cggataaaat gcttgatggt

1501 cggaagaggc ataaattccg tcagccagtt tagtctgacc atctcatctg taacatcatt

1561 ggcaacgcta cctttgccat gtttcagaaa caactctggc gcatcgggct tcccatacaa

1621 tcgatagatt gtcgcacctg attgcccgac attatcgcga gcccatttat acccatataa

1681 atcagcatcc atgttggaat ttaatcgcgg cctcgagcaa gacgtttccc gttgaatatg

1741 gctcataaca ccccttgtat tactgtttat gtaagcagac agttttattg ttcatgatga

1801 tatattttta tcttgtgcaa tgtaacatca gagattttga gacacaacgt ggctttcccc

1861 cccccccctg caagcggccg cttgcaggtc gaggaattcg ccacacccca cacctaaccc

1921 ttaaccctta accctaaccc taaccctaac cttctaaccc taaccctaac cctaacccct

1981 aaccctaacc ctaaccctaa ccctaaccct aaaccctaac cctaacccta cccctacccc

2041 tacccctaac cctaccccta accctacccc tacccctacc cctaccccta cccctgaccc

2101 tgaccctgac cctaacccta accctaaccc taaccctaac cctaacccta accctacccc

2161 tacccctacc cctccctaac cctaacccta accctgaccc taaccctaac cctaacccta

2221 accctaaccc taaccctaac cctgacccta cccctacccc tacccctgac cctacccctg

2281 accctacccc tgaccctgac cctaacccta accctaaccc taaccctaac cctaacccta

2341 aaccctaacc ctaaccctaa ccctaaccct aaccctaacc ctaaccctaa ccctaaccct

2401 aaccctaagg caactccacc ctcgcaaagg cggcgcgggg gttcgaatta ccctgttatc

2461 cctagatacg tctgcttttt gttgacttcc attgttcatt ccacggacaa aaacagagaa

2521 aggaaacgac agaggccaaa aagctcgctt tcagcacctg tcgtttcctt tcttttcaga

2581 gggtatttta aataaaaaca ttaagttatg acgaagaaga acggaaacgc cttaaaccgg

2641 aaaattttca taaatagcga aaacccgcga ggtcgccgcc ccgtaacaag gcggatcgcc

2701 ggaaaggacc cgcaaatgat aataattatc aattcctagg ctcactgata catctggcac

2761 ctactttata ataatacttt attgaaatac tttatagata ctatattcaa acatcctctc

2821 tcccattatt ttagacgcag ttcaatggca aaaactacaa ccatatctaa accacctctc

2881 agttacttaa aacctcctat gcctttttaa ccctaagaat ggtttcccac tcgtgtcgca

2941 tgttctccat cctttcccag cattcggcgc ccagttcctc gaatctcacg atatcttcct

3001 tggtcacgtc tttagcgcct ctctttcgcc cgatggtctt cctgaaaaca ccaacagcgg

3061 tgtccgcttc aagcgccgcc gcatcctcga ctttgccctc ccaaccccag ccttcaactt

3121 ctgtcttctt aacttcgacc tgcatgtgac ggttcgagct gctgccccac ttgatgcgct

3181 ctgcacctag ttcatcaagc gcagattgct cttggagcgc aatccaaaca ggggatttgg

3241 caccggtata gctgtctgtg ttgtgccagg gcgaaccaat ccagcgactg atcagaagag

3301 caagttcata tgcccacatc agaaaccagg gaactgggaa tagggtactg gcaacaatac

3361 ccggatgggt gagatacatg cgcggtcgaa tgggtttatc gcgggcctcg ctggggtcgt

3421 cgggggtgaa gaagcggctg gaagatggca tggctgcggg gagagttgca gtgagggaga

3481 gaatgtcggt gactcgttta gcggattcgt acgggccttt accattgaag cactggaagt

3541 cggacatatc aagaacactg tcgacggcct caagactgct ggaccagaca agacggccgg

3601 gtgtttcggt ttcggatcgc cgactaagta gtggaagaag ttcatgggcc aatatataat

3661 gaccaaaaac acaggctgta aacacctcgc cgagtaaagg ttccttgggc tgcattcagt

3721 caatgggtga aatcattgag ttactgcgac tgactcacat agttgtagtt gcgcttttcg

3781 ttcaagagag cagtagggag ggccatcttg aagtttggcc aagtaacaga ctgcaccaac

3841 ccctgggtca agatagtcca tacagctttg ggataattga cccctgacca tccaccatac

3901 gcagcattga atactacagt atccagcctg ggaatgcgta catttcgtag atattcgccc

3961 tgcagcccct cagggttgct caccggacct cgtaacaaag catttgcgaa agaatacacc

4021 ccgcgcaagt cacacaggtc aagctgcaag ctcagaacat ggattcgcgc aatggtatct

4081 tcccaacgat atgaactccc agcccgcgaa cgtagtgctg tagaagattg cgcagccttg

4141 ttggcgtagc cacgaagggt ttggatcgtt tgtaaggact tggacttgga tcgtgtggta

4201 gggatgagga tgaggtggga gcgcaaagag cgtgtagcaa ggaattcgtc gataagtcgt

4261 tctccaatac tgagaccgat accacttgag ttgcattagc tgtcgctccc tatatatagc

4321 ctggagcttt tgaacaaacc tattggcacc tgtgatcaga acaaaaagtt gttcctgacc

4381 cgagactccc tcccaggggg ctgtcgccgg cgacaattgg gagcccattt ggatgcttgg

4441 gtagaatagg taagtcagat tgaatctgaa ataaagggag gaagggcgaa cttaagaagg

4501 tatgaccggg tcgtccactt accttgcttg acaaacgcac caagttatcg tgcaccaagc

4561 agcagatgat aataatgtcc tcgttcctgt ctgctaataa gagtcacact tcgagcgccg

4621 ccgctactgc tacaagtggg gctgatctga ccagttgcct aaatgaacca tcttgtcaaa

4681 cgacacaaat tttgtgctca ccgcctggac gactaaacca aaataggcat tcattgttga

4741 cctccactag ctccagccaa gcccaaaaaa tggcgcgcca tatggtacgt aaaacgacgg

4801 ccagtgagcg cgcgtaatac gactcactat agggcgaatt gggtactcaa attggttata

4861 aatgtaggta ttacctgtac attttattta ttcgagaaaa acaaacaaac caaaacgatg

4921 cagcgatcac taccggcgtc atccggaatt gaaacggcgc gcgttgaggg gggccttctt

4981 gttcggacac acacggggcc gccaaagtgg ggcccatacg gctgcccatc ccttgctctc

5041 caataccctg gtcatgtgac tcgaaattgg agactcacaa agatgctttg aaaccccgcc

5101 gggaactttt tctcacagac acaatcagtc gaccttatca tcgcaaaatc gacaaacctc

5161 aaaaagaact cgaagagtca gtctcccttg cggttaaact gattgatatc tcgtgttctc

5221 taaaccttca ataagtgagt tactgcgcac aaagccgact atactgagca ccaacgcgaa

5281 ttgctcacgt tgtcgcctaa cagatcttgg ctttcgtagg aacccaatct tcaaagcggc

5341 cgcctcgagg gtacatggat gacaggatac aagacattta caaaaagatc gagcgcgaga

5401 aagctttgat aaatgctgcc aacttgatgc gacagcagac caacaatgat gccgtccgat

5461 ccaagctcga cacccagatg cgcgagggcc gccgcaacct cgagttcttt gaagggacac

5521 tgcgggaaat gcagatgcgc agtatgggtc aaggaatgga caacctgagc atcggcgggt

5581 ccacccttgc cgccagcgcc tcctcttcgt cccgcccgcg aagtgccgtc gaggaagatg

5641 gccccatgcc tcctcctaaa gatgggggat atggcgctgg tggcgacggc tatggccaaa

5701 cgcagtacag tcagatcggc gagcatggcg atctgatgcc gcctcgaggt cctttcgcca

5761 accaaggccc agggagccat attccaaaaa caagaccaaa ctttacaaag ctgggtgagt

5821 cgaccggttg caataactag cggcaatgta aacatcataa gattccctag ctgacaactg

5881 caatttgtat cgcgacagat ctgatcaaat acgacacgcc ataccttggc ccccggattc

5941 agctcatgct gtcgcaaatt caattcaaac tcaatgttga ggaacagtat ctgaaaggca

6001 tcgaaaagat ggtacagttg tatcagatgg agggtgataa gaagagcaga gccgatgcag

6061 ccgcccgtcg ggttgagagc aagcaaaaaa tcgtactatt gaaacaggct ttgcgaagat

6121 acgaagaatt gcatatagac attgactcgg gagattcacc ggatggtaag catttggcgc

6181 tggaaggtca ctacccctct gggctcaatt acagtacctg ctaacacttt tactttcccg

6241 cagatgatag tatcaacatg cccaacctcc ggaagcccct taccggtcaa ttatcgattc

6301 gagtcattgc catcaaagat gtcgaccacg ctccgacagg gcgctttgcc cgaggccccg

6361 acactttcgt ggcgatcaag gtggaagaca atatagctgc ccgtaccaga gtatctcgaa

6421 cagacaggtg ggagaacgag tatcacaacg tcgaggtcga caaggcaaac gaaattgagc

6481 tcaccgttta cgacaaaccc ggagagcact caatccccat cgggatgctt tgggtcagaa

6541 tatctgatat cgctgaagag ctgcgaagga agaagatcga agcggaaact aatagtctgg

6601 gctgggtgtc agcggaccgc atgggcgacg tgaaccctgc tgccagagtt ccccctccgc

6661 aattcccaat gaacactcaa cagcagcagc ctggtggtcc agtatccccc ggcatccagg

6721 gccagcagca agggtatggt caaccggggg atccgcagca accgggtcag caaggtcaga

6781 tgggacaggt tcaggaacct attacaggct ggtttaacct cgagcctact ggctccattc

6841 agctcagctt gaacttcttc aaacagagca aggaccgtcg ccctgtggac ttgggtcttg

6901 gacgcaaggg cgccatccgc caacgcaagg aggaggttca cgagatgtat ggacacaagt

6961 ttgtggagcg ccagttctac aacatcatgc gctgcgctct ctgcggagac ttcctcaagg

7021 gcggatcggg gatgcagtgc gaggattgca agtacacttg ccatatcaag tgctacacga

7081 gcgtggtcac caaatgcatc agcaaatcca acgctgaaac tgatccggat gaggagaaga

7141 tcaaccaccg gattcctcac agattcgtgg cattctctaa cctcactgct aattggtgct

7201 gtcactgcgg ttacctattg ccattcggaa agaaaaactg cagaaaatgc agcgagtgtc

7261 aaatggcggc ccatgctggc tgtgtacatc tggtccctga cttttgcggt atgtcgatgg

7321 ctgtcgcgaa tcaaattctc gagggtatca ggtcacagaa acaacgccag caaaaggcaa

7381 gctctatgag tgaccgcacg ttgcgcagtg gaaaaatgag cccgcctggc tccgggcatg

7441 cctcatcggc atcattctct cagggcatgg gatcaagcta cggtcaggca tctcctgaag

7501 ccacggaagc tgccaaattc atgtactcta gccaaacttc acctcagcgg atcacctccc

7561 cagacaggac atcaagctcg tcacaagctg cggctgcggc aacggctgct atgaccggtc

7621 tgccgggagc catgtcttct cagcctggac gacaaggaca gcagccaata actgactacg

7681 gttcggccag cgggggtcgc tatggttcgt atggtccgca cgatgatcca tacgcacagc

7741 cacagcaatc gccaccgccg caacagcaag cggcttacgg acagcctcag cagcgaaagt

7801 acaaccccgc ggactatgcc aacatcagcg ggggatacgg cagtcagccg cagatggctc

7861 agcaaccgca gcaagctcgg ccccaacagc agcagcaaca gccgttgtac tctccacagc

7921 agcacgcatc ccaagctcag caacccctgt cacctgttaa gcagcagcat caggaacaac

7981 aaatcatctc accgactgca ggaaccgtca ttccgacatc tgcgaagagg cctcttcctt

8041 cagccacgga tcctggtaca ggccagcgta tcgggcttga ccacttcaac ttcttggctg

8101 tgcttggaaa gggcaatttc ggcaaggtca tgcttgcgga atccaagaag actaggaaac

8161 tctatgccat caaagtcctc aagaaggagt tcattatcga gaacgacgag gttgagagca

8221 tccgctcaga gaagcgggtg ttcctcatcg ccaacaggga acggcatcca ttcctcacca

8281 acctgcacgc ctgtttccag accgagacgc gtgtatactt cgtggaagaa tacattagtg

8341 gtggagattt gatgttgcac attcaacgag gacagttcgg cacccgtagg gcccagtaag

8401 tttacaaaac cacagtggcc cctgccacac cgtgaaagtt ttcactgaca ctccgtaggt

8461 tctatgctgc cgaagtttgc ttggctctca agtacttcca cgagaacggt gtcatctacc

8521 gtgatctcaa gctcgacaac atccttctca ctcttgacgg tcacatcaag attgccgact

8581 atggtctttg caaggaagac atgtggtacg gctctaccac gagcacgttc tgcggtaccc

8641 ccgagtttat ggcaccagaa gtaagcaaat atggtcccct agcttttgac cagtaactga

8701 cttgcgcgcg cagattttgc ttgacaagaa gtatggccga gcagtcgact ggtgggcgtt

8761 tggcgttttg atctaccaga tgcttcttca gcagtcgcct ttcagaggtg aagacgaaga

8821 tgagatctac gacgccattc tggcggacga gccgctgtat cctattcaca tgccgcgcga

8881 ctctgtttcg atcttgcaga agctgctcac cagggaaccg gaccagcgtc tgggaagtgg

8941 cccgactgat gcccaggagg tcatgtcgca gcccttcttc cgcaacatcg tctgggatga

9001 catctaccac aagcgcgtgg cgccaccgtt cctgccgcag atcaagagtg ccacggacac

9061 gagtaacttt gactcagagt ttactagtgt tacgcctgtc ctgacgccag tccaatcagg

9121 taagtagcca aggccgtatg acgaactgct ttgatatcag caactaacca gtatcttctc

9181 aatacagttc tgtcccaagc gatgcaggag gagttccgcg gcttttcgta cacggcagac

9241 tttgattgat gctgcctcct ccgaggacgt catcaaggag ttcatgcgct tcaaggtgcg

9301 catggagggc tccgtgaacg gccacgagtt cgagatcgag ggcgagggcg agggccgccc

9361 ctacgagggc acccagaccg ccaagctgaa ggtgaccaag ggcggccccc tgcccttcgc

9421 ctgggacatc ctgtcccctc agttccagta cggctccaag gcctacgtga agcaccccgc

9481 cgacatcccc gactacttga agctgtcctt ccccgagggc ttcaagtggg agcgcgtgat

9541 gaacttcgag gacggcggcg tggtgaccgt gacccaggac tcctccctgc aggacggcga

9601 gttcatctac aaggtgaagc tgcgcggcac caacttcccc tccgacggcc ccgtaatgca

9661 gaagaagaca atgggctggg aggcctccac cgagcggatg taccccgagg acggcgccct

9721 gaagggcgag atcaagatga ggctgaagct gaaggacggc ggccactacg acgccgaggt

9781 caagaccacc tacatggcca agaagcccgt gcagctgccc ggcgcctaca agaccgacat

9841 caagctggac atcacctccc acaacgagga ctacaccatc gtggaacagt acgagcgcgc

9901 cgagggccgc cacagtactg gagcatgaat cattccactc aacattcagg ctcctctgcg

9961 cacgtaaagt gccaaaggca ataccctgct cggtggaatg ccgccgggct tgtcgatttt

10021 acgcacatat gcgcattctt gacttgaagc ggaggagttc ttcgttgcgg gttacagtgt

10081 tttaataaaa gaatggtcaa atcaaactgc tagatatacc tgtcagacac tctagttgtt

10141 gacccctata ctcttaatac atcagacagt acatgcatgt tgcatgatga tgatatgtct

10201 gtttagattc cagtgtctac tgctggccta gtttctcggt actatgcata gatcctacat

10261 gcatgtgagc aaaaggccag caaaaggcca ggaaccgtaa aaaggccgcg ttgctggcgt

10321 ttttccatag gctccgcccc cctgacgagc atcacaaaaa tcgacgctca agtcagaggt

10381 ggcgaaaccc gacaggacta taaagatacc aggcgtttcc ccctggaagc tccctcgtgc

10441 gctctcctgt tccgaccctg ccgcttaccg gatacctgtc cgcctttctc ccttcgggaa

10501 gcgtggcgct ttctcatagc tcacgctgta ggtatctcag ttcggtgtag gtcgttcgct

10561 ccaagctggg ctgtgtgcac gaaccccccg ttcagcccga ccgctgcgcc ttatccggta

10621 actatcgtct tgagtccaac ccggtaagac acgacttatc gccactggca gcagccactg

10681 gtaacaggat tagcagagcg aggtatgtag gcggtgctac agagttcttg aagtggtggc

10741 ctaactacgg ctacactaga aggacagtat ttggtatctg cgctctgctg aagccagtta

10801 ccttcggaaa aagagttggt agctcttgat ccggcaaaca aaccaccgct ggtagcggtg

10861 gtttttttgt ttgcaagcag cagattacgc gcagaaaaaa aggatctcaa gaagatcctt

10921 tgatcttttc tacggggtct gacgctcagt ggaacgaaaa ctcacgttaa gggattttgg

10981 tcatgagatt atcaaaaagg atcttcacct agatcctttt aaattaaaaa tgaagtttta

11041 aatcaatcta aagtatatat gagtaaactt ggtctgacag ttaccaatgc ttaatcagtg

11101 aggcacctat ctcagcgatc tgtctatttc gttcatccat agttgcctga ctccccgtcg

11161 tgtagataac tacgatacgg gagggcttac catctggccc cagtgctgca atgataccgc

11221 gagacccacg ctcaccggct ccagatttat cagcaataaa ccagccagcc ggaagggccg

11281 agcgcagaag tggtcctgca actttatccg cctccatcca gtctattaat tgttgccggg

11341 aagctagagt aagtagttcg ccagttaata gtttgcgcaa cgttgttgcc attgctgcag

11401 gcatcgtggt gtcacgctcg tcgtttggta tggcttcatt cagctccggt tcccaacgat

11461 caaggcgagt tacatgatcc cccatgttgt gcaaaaaagc ggttagctcc ttcggtcctc

11521 cgatcgttgt cagaagtaag ttggccgcag tgttatcact catggttatg gcagcactgc

11581 ataattctct tactgtcatg ccatccgtaa gatgcttttc tgtgactggt gagtactcaa

11641 ccaagtcatt ctgagaatag tgtatgcggc gaccgagttg ctcttgcccg gcgtcaacac

11701 gggataatac cgcgccacat agcagaactt taaaagtgct catcattgga aaacgttctt

11761 cggggcgaaa actctcaagg atcttaccgc tgttgagatc cagttcgatg taacccactc

11821 gtgcacccaa ctgatcttca gcatctttta ctttcaccag cgtttctggg tgagcaaaaa

11881 caggaaggca aaatgccgca aaaaagggaa taagggcgac acggaaatgt tgaatactca

11941 tactcttcct ttttcaatat tattgaagca tttatcaggg ttattgtctc atgagcggat

12001 acatatttga atgtatttag aaaaataaac aaataggggt tccgcgcaca tttccccgaa

12061 aagtgccacc tgacgtctaa gaaaccatta ttatcatgac attaacctat aaaaataggc

12121 gtatcacgag gccctttcgt cttcaagaat tcgcggcccc gcatgggccc a

//
